# Geometric network analysis provides prognostic information in patients with high grade serous carcinoma of the ovary treated with immune checkpoint inhibitors

**DOI:** 10.1038/s41525-021-00259-9

**Published:** 2021-11-24

**Authors:** Rena Elkin, Jung Hun Oh, Ying L. Liu, Pier Selenica, Britta Weigelt, Jorge S. Reis-Filho, Dmitriy Zamarin, Joseph O. Deasy, Larry Norton, Arnold J. Levine, Allen R. Tannenbaum

**Affiliations:** 1 Department of Medical Physics, Memorial Sloan Kettering Cancer Center, New York, NY 10065 USA; 2 Department of Medicine, Memorial Sloan Kettering Cancer Center, New York, NY 10065 USA; 3 Department of Pathology, Memorial Sloan Kettering Cancer Center, New York, NY 10065 USA; 4 Institute for Advanced Study, Princeton, NJ 08540 USA; 5 Departments of Computer Science and Applied Mathematics & Statistics, Stony Brook University, Stony Brook, NY 11794 USA

**Keywords:** Cancer genomics, Prognostic markers, Bioinformatics

## Abstract

Network analysis methods can potentially quantify cancer aberrations in gene networks without introducing fitted parameters or variable selection. A new network curvature-based method is introduced to provide an integrated measure of variability within cancer gene networks. The method is applied to high-grade serous ovarian cancers (HGSOCs) to predict response to immune checkpoint inhibitors (ICIs) and to rank key genes associated with prognosis. Copy number alterations (CNAs) from targeted and whole-exome sequencing data were extracted for HGSOC patients (*n* = 45) treated with ICIs. CNAs at a gene level were represented on a protein–protein interaction network to define patient-specific networks with a fixed topology. A version of Ollivier–Ricci curvature was used to identify genes that play a potentially key role in response to immunotherapy and further to stratify patients at high risk of mortality. Overall survival (OS) was defined as the time from the start of ICI treatment to either death or last follow-up. Kaplan–Meier analysis with log-rank test was performed to assess OS between the high and low curvature classified groups. The network curvature analysis stratified patients at high risk of mortality with *p* = 0.00047 in Kaplan–Meier analysis in HGSOC patients receiving ICI. Genes with high curvature were in accordance with CNAs relevant to ovarian cancer. Network curvature using CNAs has the potential to be a novel predictor for OS in HGSOC patients treated with immunotherapy.

## Introduction

Facilitated by advances in genomic sequencing techniques and the ongoing development of highly curated protein–protein interactome (PPI) databases (e.g., Human Reference Protein Database (HPRD,^1,2^), The Human Reference Interactome^3^, Search Tool for the Retrieval of Interacting Genes/Proteins^4^), we adopt a network approach to investigate biological features pertaining to overall survival (OS) in ovarian cancer (OC) based on copy number alterations (CNAs) in tumor tissues. The past decade has seen a large rise in the development of methods for analyzing large, complex networks, as exhibited by the rapidly growing literature. We draw on geometric notions to inform about the network structure, defined by evidence-based interactions provided by the PPI. Our network analysis methodology is unsupervised without fitting parameters or feature selection and is not constrained to the underlying topology alone. Indeed, since cancer has been demonstrated to exhibit functional robustness in connection to geometric properties of its network representation^5^, we utilize Ollivier’s discrete notion of Ricci curvature on weighted graphs, referred to as Ollivier–Ricci (OR) curvature^6^.

The focus of this paper is to introduce a geometric network method for cancer with the key application to high grade serous ovarian cancer (HGSOC). Biomarkers of response to immune checkpoint blockade in HGSOC remain largely unknown. Unlike non-small cell lung cancers and melanomas that exhibit increased immunogenicity due to high tumor mutational burden (TMB)^7–11^, HGSOCs exhibit low TMB^12^. In virtually all cases, HGSOCs are a disorder of loss of function gene mutations (*TP53*) leading to CNAs, and subsequently resulting in overexpressed copy number in multiple genes including oncogenes (e.g., *KRAS*, *MYC*, *CCNE1*, and *AKT1*) commonly due to aneuploidy^13,14^. The impact of these alterations on response to immunotherapy is unknown; furthermore, it is unlikely that individual pathway alterations would be strongly predictive. This manuscript develops a mathematical method that constructs a network of these gene pathways where each node (gene) is quantitated by CNAs and for each tumor, the changes in the architecture or connectivity of the network are measured by a parameter termed *curvature* of the edges of the network. Curvature measures the connectivity in the sense of feedback loops, and the copy number measures the abundance of each node and its projected impact upon the changes in the network architecture. (More rigorous details about this will be given in the Methods Section.) Nodal curvature may exhibit more variation than the CNAs, reflecting the integration of the gene copy numbers and the local impact of their alteration on the network. Thus, curvature has the potential to differentiate responders from non-responders in patients treated with immune checkpoint inhibitors (ICIs) that could not be predicted from a single gene alone. Note that in this paper, gene names will be italicized in the main text, except in the tables for easier reading.

Curvature is a local measure of how a geometric object (e.g., curve, surface, space) deviates from being *flat* in the Euclidean sense. While the physical interpretation of curvature in 3-dimensional Euclidean space is a familiar concept, intuition for curvature as a rigorous mathematical concept is often elusive, as the mathematical theory is not bound by the same physical constraints. This allows for curvature to be generalized to continuous spaces of higher dimensions (classically, Riemannian manifolds), and even to discrete spaces (Supplementary Fig. 1). The mathematical construct, however, is not solely of abstract, theoretical value. The archetypical example is the curvature of space-time which was integral to Einstein’s theory of general relativity. Although perhaps less intuitive, the geometric insight that curvature provides is applicable to other physical phenomena. In particular, change in OR curvature^6^ has a strong mathematical connection to changes in robustness via change in entropy. Note that we are using *change in curvature* in the sense as a difference in curvature Δ*κ* between networks. This is a remarkable result facilitated by the theory of optimal mass transport (OMT) attributed to Sturm, Lott, and Villani^15,16^. The change in OR curvature has previously been used as an effective quantitative proxy for the qualitative notion of changes in robustness in various types of networks^5,17^. In the present work, we employ curvature to predict patient survival and investigate primary components of functional robustness to identify key genes contributing to functional dysregulation in HGSOC.

Various biomarkers including PD-L1 and the spatial distribution and composition of the immune microenvironment are being investigated in the context of response to ICI^12^, but the present work focuses on extracting information from gene-level information. It is becoming more apparent that the use of genomic data (e.g., mutations, gene expression, CNAs) with the corresponding functional network representation can provide more insights into understanding the underlying biology of cancer. Thus, graph-based tools may be more powerful for investigating complex genomic networks than methods that aim to analyze and quantify the data independently.

Genomic networks have a topology (i.e., a connectivity structure), but they also have a geometry, i.e., curvature, which gives a measure of their functional robustness. Graph curvature is intimately related to the number of invariant triangles, i.e., *feedback loops* at a given vertex, and the curvature between two vertices describes the degree of overlap between their respective neighborhoods^18^. Informally, graphs with positive curvature characteristically contain many triangles (redundant feedback loops), contributing to its functional robustness with respect to a damaged or deleted edge. The more neighbors two given nodes have in common (i.e., triangles), the easier it is for information to flow between them. By weighing the ease with which information can be transferred from one node to another against the ground distance between them, curvature provides a local measure of functional connectivity compared to ordinary measures of connectivity which identify hubs based on degree. We show not only that the total curvature of a network can be used to predict overall patient survival in HGSOC, but it is also more effective than standard clinical parameters such as TMB.

Typically, the curvature is computed on a network using the standard hop distance (where every edge in a path connecting two nodes is treated as a hop) with node weights that are continuous in nature (e.g., gene expression). Here, we use a *weighted hop distance* derived from the data as the underlying graph metric, so the distance between two nodes depends not only on the topology, but on the likelihood of interaction as well. Using node weights assigned by (discrete) CNAs, we show that curvature may also be informative in the discrete data setting. Furthermore, we show that the network topology without any additional information may be used as a reference to identify potential key players responsible for the functional robustness, even when limited data is available, as demonstrated in this study. Top identified genes such as *TP53*, whose known aberrant functional behavior has been attributed as a leading influence in the development/progression of ovarian cancer^19^, serve as validation for the proposed methodology.

Specifically, we create a shared topology, but with sample-specific gene interaction networks. The interactions are taken from the HPRD, where the protein interactions are assumed to serve as a proxy for the underlying gene interactions. We then supplement topology (i.e., connectivity) with sample-specific node weights taken to be the given copy number data. For each network, curvature is then computed at three scales: on edges, nodes, and the entire network. Analogous to Ricci curvature defined on tangent directions at a point on a Riemannian manifold and its contraction *scalar curvature* defined on the points of the manifold, the formulation of OR curvature is computed on all edges in the network and scalar curvature is computed on all nodes by contracting the OR (edge) curvature with the invariant distribution associated with the weighted network^6^. The *total curvature* of the network is then computed by contracting the scalar curvature to a single scalar. (See Eq. (9) for the precise definition.)

## Results

### Survival analysis

The prognostic value of the total curvature *κ*_*G*_ in Eq. (12) and standard genomic parameters including TMB, the fraction of genome altered (FGA) and large-scale state transition (LST) scores (representing homologous recombination deficiency status) were assessed with respect to the HGSOC cohort (*n* = 45). For each parameter (*κ*_*G*_, TMB, FGA, LST), the cohort was stratified into two groups according to the 25th percentile (low vs. high) of individual values. The cutoff was selected based on the location where the curve fitted to the sorted total curvature values starts slowly incrementing and is approximately linear (Supplementary Fig. 6). An alternative cut point using maximally selected log-rank statistics^20,21^ was assessed as well and resulted in a comparable split (Supplementary Fig. 7). However, a larger cohort is needed for further validation. The effectiveness of each parameter in terms of OS was evaluated using the Kaplan–Meier (KM) analysis^22^.

**Fig. 1:**
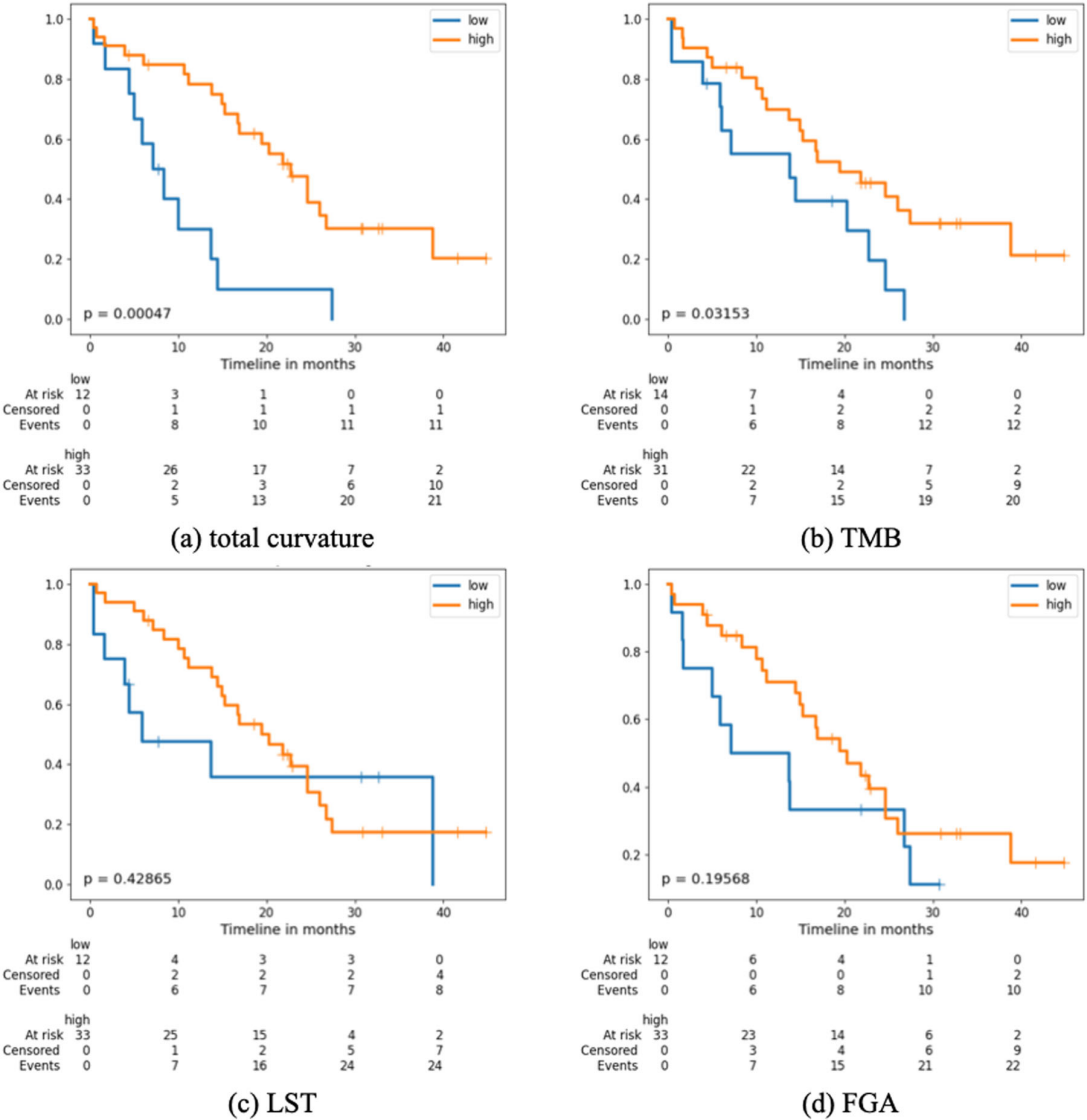
Survival curves for HGSOC samples (*n* = 45) stratified low and high groups by the 25th percentile of total curvature and genomic parameters. *P* values were derived from the log-rank test.

OS was defined from the start of immunotherapy treatment until either death or last follow-up^12^. Survival curves for each parameter were plotted according to the KM estimator, shown in Fig. 1 along with the corresponding log-rank *p* values (total curvature: *p* = 0.00047; TMB: *p* = 0.03153; LST: *p* = 0.42865; FGA: *p* = 0.19568). While both TMB and total curvature *κ*_*G*_ were found to be significant factors in predicting patient survival, the p-value for total curvature was almost 2 orders of magnitude smaller as compared to TMB, whose p-value was just marginally significant. The effective prognostic predictive power of the total curvature, particularly in comparison to the genomic parameters, is one of the major contributions of this work. See Supplementary Material for validation (Supplementary Figs. 4 and 5) and survival analysis on the metastasis subcohort (Supplementary Fig. 9).

In order to assess that the prediction is not independent of receiving immunotherapy treatment, we repeated the curvature and survival analysis pipeline on IMPACT data from HGSOC samples that did not receive ICIs. It is interesting to note that total curvature was not predictive of survival in this setting (Supplementary Fig. 8), highlighting that our findings may be immunotherapy-specific. However, it is also important to point out that OS was defined from the time of diagnosis for the analysis of this data set, whereas in the analysis of 45 HGSOC patients treated with ICIs, OS was defined from the start date of immunotherapy, and all 45 patients had recurrent tumors with a substantial time gap between the time of first diagnosis and the start date of immunotherapy. Lastly, no statistically significant differences were found using progression-free survival (PFS) in this cohort. This is not novel and a number of studies have increasingly demonstrated the ability of ICI to impact OS without significant impact on PFS. We have previously demonstrated that ICI therapy may positively influence responses to subsequent chemotherapy^12^, suggesting that ICI may positively impact disease biology without immediate apparent clinical benefit.

### Functional biomarkers

Genes that exhibit large changes in scalar curvature are identified as the genes that potentially play a key role in altering the network robustness (i.e., functional connectivity). This requires a reference for comparison, typically using data collected at a reference time (e.g., after immunotherapy treatment) or data collected from a reference sample (e.g., normal tissue). Often no such reference data are available, as was the case here where CNA data from only one time point were provided. Considering the distinction in survival curves obtained via curvature, we therefore used the high and low-risk groups (as previously defined by the 25th percentile of the total curvature and dichotomized into low and high curvature groups, respectively) for points of comparison. Genes were ranked by the difference in average scalar curvature between the low and high-risk groups (Δ*κ*_risk_). The change in curvature measures the relative gene implication in the stabilization (or de-stabilization) of local network robustness driving changes in feedback connectivity pertaining to survival. Since both increased and decreased functionality is of interest, the top 50 ranked genes that exhibited the largest positive (Δ*κ*_risk_ > 0) and largest negative (Δ*κ*_risk_ < 0) change in curvature, yielding 100 candidate genes associated with risk, are listed in Table 1 (and listed alphabetically in Supplementary Table 5).

**Table 1:** Changes in average scalar curvature based on risk (low vs high).

Rank	Gene	Δ*κ*_risk_ > 0	Gene	Δ*κ*_risk_ < 0
0	TP53	0.208647	CREBBP	−0.064223
1	ATXN1	0.102823	SHC1	−0.031456
2	EP300	0.044184	PTK2	−0.026202
3	SMAD2	0.042756	AR	−0.025316
4	PIK3R1	0.037015	MYC	−0.022608
5	SRC	0.033112	JUN	−0.019546
6	SMAD4	0.032177	LYN	−0.011148
7	RB1	0.031043	YWHAQ	−0.010984
8	ESR1	0.027914	GSK3B	−0.009017
9	PRKCA	0.027253	STAT1	−0.008248
10	CTNNB1	0.025121	CDK5	−0.007480
11	GRB2	0.016848	FN1	−0.006947
12	YWHAE	0.015125	COPS6	−0.006251
13	DLG4	0.014966	SMAD3	−0.006133
14	PRKCD	0.014742	PAK1	−0.006091
15	ACTB	0.013456	MYOC	−0.005464
16	EWSR1	0.012300	SMURF1	−0.005438
17	TGFBR1	0.010799	SUMO1	−0.004455
18	RAC1	0.008937	PARP1	−0.004274
19	PLCG1	0.008423	CRMP1	−0.004271
20	CHD3	0.007997	HSF1	−0.004155
21	DVL2	0.007476	HIPK2	−0.004038
22	BCL2	0.007009	CDC42	−0.004017
23	RANBP9	0.006879	POU2F1	−0.003838
24	MAPK1	0.006630	ACVR1	−0.003651
25	POLR2A	0.006468	HTT	−0.003537
26	CRK	0.006375	JAK1	−0.003520
27	APP	0.006256	PDPK1	−0.003497
28	PCNA	0.005935	PIK3R2	−0.003423
29	COIL	0.005350	FGFR1	−0.003352
30	MAPK14	0.005097	CDKN1A	−0.003205
31	NR3C1	0.004981	MAGEA11	−0.003165
32	AKT1	0.004925	GNAI1	−0.003125
33	EGFR	0.004918	PRKCE	−0.003090
34	RHOA	0.004635	XPO1	−0.002919
35	RAF1	0.004159	BTK	−0.002855
36	SMAD7	0.004071	MUC1	−0.002814
37	NCOR1	0.004038	EIF2AK2	−0.002807
38	RASA1	0.003998	CASP8	−0.002758
39	FXR2	0.003879	CSNK2A2	−0.002717
40	RPA1	0.003560	MDM2	−0.002710
41	HRAS	0.003525	NTRK1	−0.002636
42	UBB	0.003302	ADAM15	−0.002541
43	BRCA1	0.003292	FASLG	−0.002522
44	SUMO4	0.003283	VIM	−0.002436
45	ARRB2	0.003248	CD247	−0.002372
46	XRCC6	0.003065	AXIN1	−0.002333
47	HGS	0.003025	SMARCA4	−0.002256
48	HDAC3	0.002965	SNAPIN	−0.002246
49	HSP90AA1	0.002924	PPP2R5A	−0.002187

Top 50 genes ranked by positive (Δ*κ*_risk_ > 0) and negative (Δ*κ*_risk_ < 0) difference in average scalar curvature between low risk (*n* = 33) and high risk (*n* = 12) groups.

Similarly, we investigated the top genes ranked by the difference in average scalar curvature between sub-groups based on available clinical data as an exploratory analysis. Of ancillary interest were the top-ranked candidate driver genes that demonstrate functional network response to ICI and their association to survival as exhibited by disparities in network robustness measured between those who were alive or deceased at last follow-up (Δ*κ*_OS_; Supplementary Table 1) and predominant changes in functional connectivity due to DNA level dysregulation that occurs between primary and metastatic tumors (Δ*κ*_PM_; Supplementary Table 2). Lastly, we used the network topology itself as a frame of reference. Treating the fixed network topology as an unweighted graph (i.e., all node weights are uniformly set to 1), we computed the scalar curvature on this reference topology network in the same manner as detailed above. This provides a measure of discordance in functional connectivity between the HGSOC network and its underlying topological structure (Δ*κ*_ref_; Supplementary Table 3). It is interesting to note that in all of the comparisons *TP53* appeared at the top of all positive changes in curvature indicating its functional centrality in HGSOC.

Substantial overlap in the top 50 (positive and negative) ranked genes was noted from all of the comparisons performed, resulting in 171 unique genes listed in Supplementary Table 4 (Supplementary Figs. 12 and 13). The choice of selecting the top 50 genes was largely arbitrary with the following rationale. The assertion that critical genes may be identified as those exhibiting larger changes in curvature is supported by the theory, but curvature is a continuous variable with no obvious cutoff. Since there is also an exploratory component to this analysis, we opted for a cutoff that would yield a manageable set of genes that reasonably included the key influential players. Out of 3489 genes in the network, this resulted in 50 (positive and negative) candidate genes. See Supplementary Fig. 10 for a further *sub-curvature* analysis on the association between the highlighted candidate genes and survival.

### Relationship between total curvature and genomic features

**Fig. 2:**
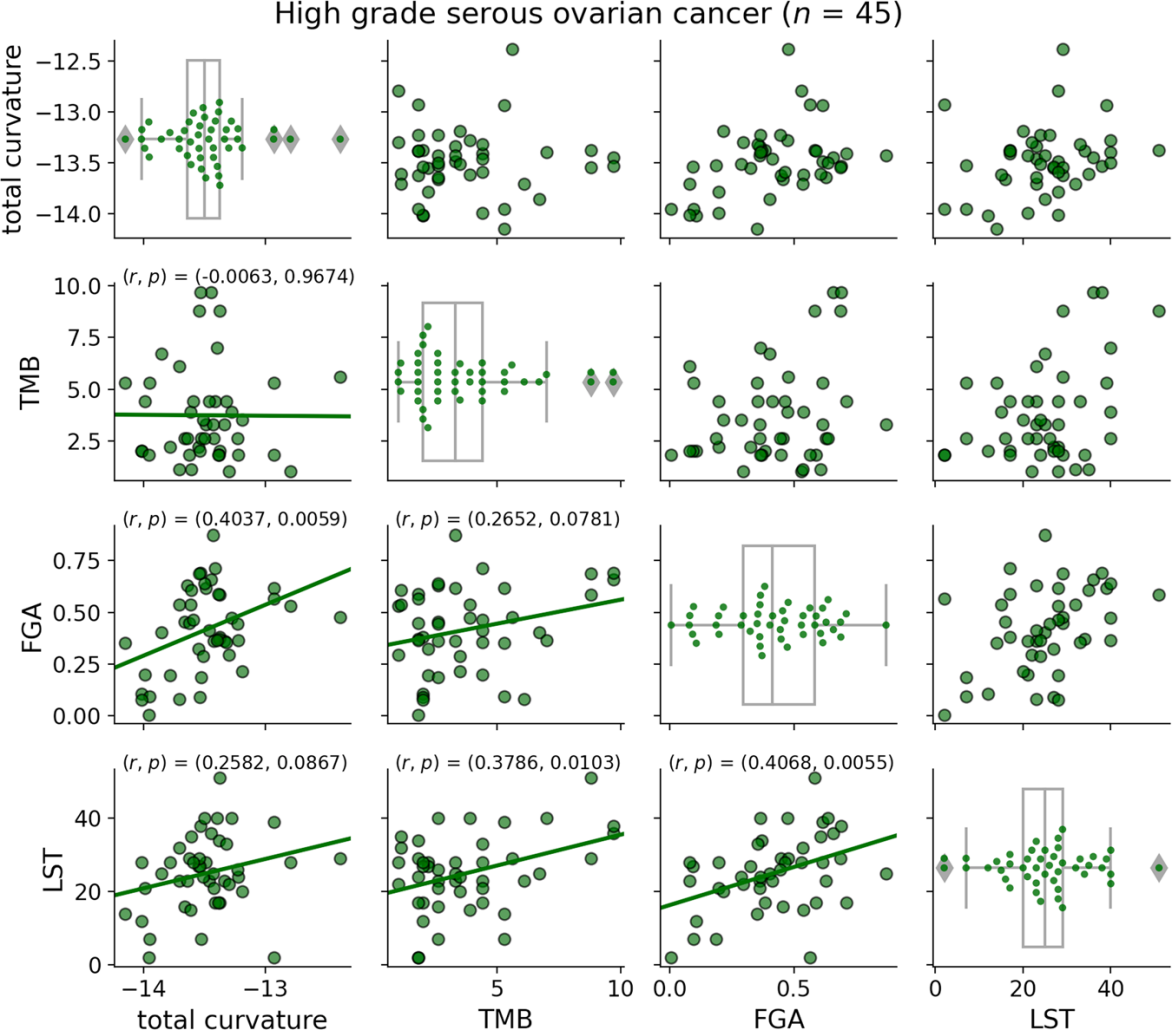
Linear regression of total curvature onto clinical parameters using HGSOC samples (*n* = 45). The lower triangle includes the regression line, Pearson correlation (*r*), and associated *p* value.

Lastly, we explored the relationship between total curvature and genomic features (TMB, FGA, LST). Linear regression analysis and Pearson correlation (*r*) with *p* values were used to assess the correlation between total curvature and each of the clinical features (TMB: *p* = 0.9674; FGA: *p* = 0.0059; LST: *p* = 0.0867). This analysis suggests that total curvature is significantly correlated with FGA. This result is not entirely surprising considering that FGA is a surrogate measure of CN changes and the curvature measures dysregulation of the CN-weighted network. However, total curvature yields high- and low-risk groups with a significant difference in survival, whereas FGA does not. The difference is that total curvature accounts for an extra level of information, namely the connectivity, that is not evident from CNAs alone. We believe this is compelling evidence that network dysregulation, as measured by curvature, has the potential to provide critical insight for analyzing immune response. More samples are needed to verify this result but it is interesting to note that further investigation into FGA as a potential biomarker for survival in HGSOC has been proposed^12^. Linear regression plots on the HGSOC cohort (*n* = 45) are shown in Fig. 2.

## Discussion

**Fig. 3:**
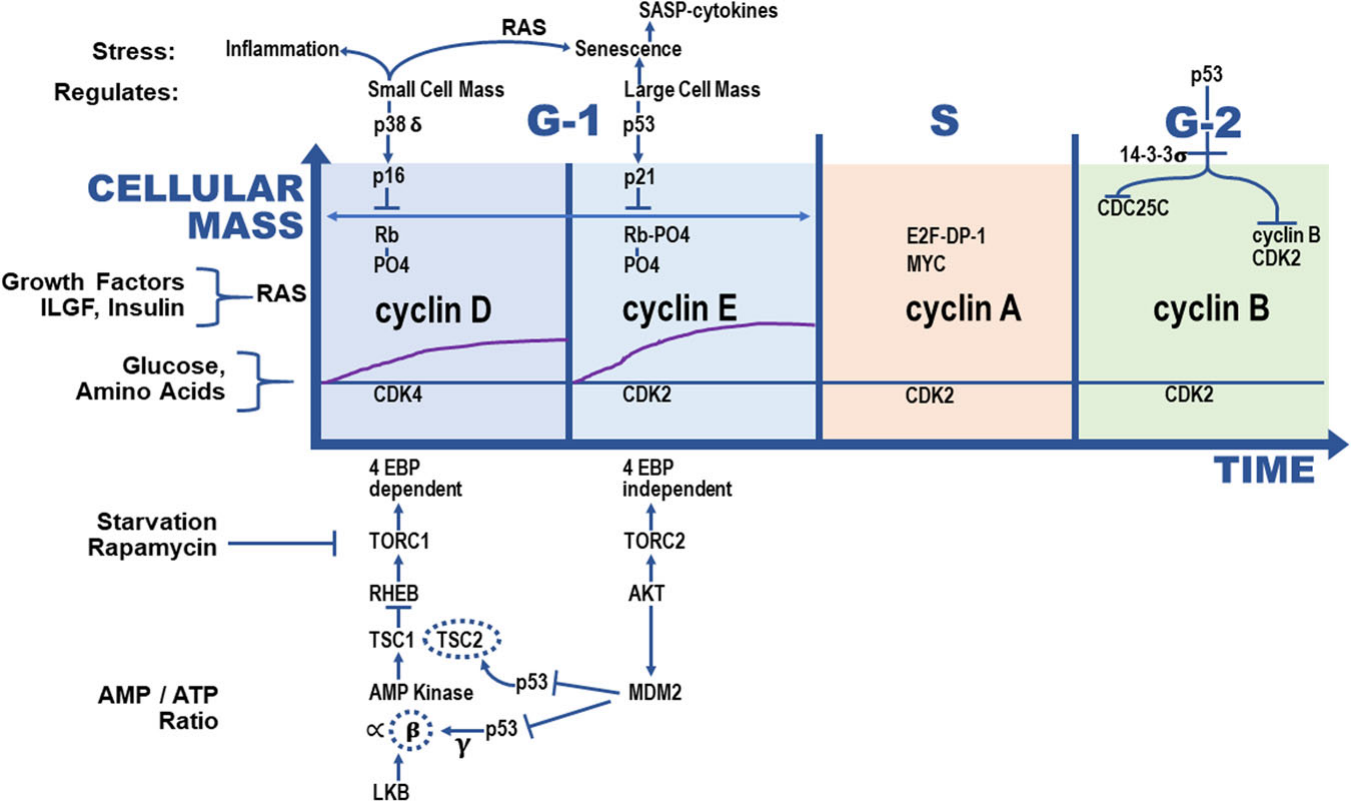
Genes involved in serous ovarian cancer in the G-1 phase of the cell cycle: the G-1 phase of the cell cycle can be divided up into cyclin D-CDK4/6 early events and cyclin E-CDK2 later events. The inhibitors of these protein kinase activities, p38 and p16 for cyclin D and p53 and p21 for cyclin E, are shown above the cyclin D and E panels. The activating pathways for cyclin D (TORC1) and cyclin E (TORC2) are shown below these panels. The mutational loss of *TP53* and the amplification of cyclin E results in the loss of control of cyclin E levels and the hyper-amplification of centrosome numbers destabilizing the copy number control of chromosome numbers (aneuploidy) and gene copy numbers. Serous ovarian cancers commonly have *KRAS*, *MYC*, and *AKT1* genes or chromosome amplifications and overexpression. The *CDKN1A* gene which codes for p21 is not mutated suggesting that it has additional functions required elsewhere for viability or that additional functions of p53 must be lost for ovarian cancers. Every gene highlighted in this figure can be found genetically altered in a cancer of other tissue types.

Mutational profiles of HGSOCs are characterized by abnormal gene CNAs, which result in protein overexpression or underexpression^13^. The majority of these OCs are characterized by inactivating mutations or loss of *TP53*, leading to aneuploidy, resulting from loss of control of centrosome numbers^23^, and selection for enhanced copy number and gene expression of selected genes controlling the cell cycle (Fig. 3). These OCs commonly overexpress the cyclin E protein due to loss of p53 function, resulting in downregulation of p21 (the inhibitor of cyclin E-CDK2 activity), as well as amplification of cyclin E^13^. In addition, the serous OCs have one or more of the *KRAS*, *MYC*, and *AKT1* genes overexpressed in the late G-1 phase of the cell cycle (see Fig. 3). The *KRAS* activity signals that the cell is stimulated by growth factors and should progress through the cell cycle, the *MYC* gene regulates the transcription of hundreds of genes for cell growth and division, and the *AKT1* gene promotes TORC2 activity for entry into S-phase and stimulates AKT kinase to enhance the MDM2 E3 ubiquitin ligase to increase the destruction of the p53 protein^24^. All of these driver gene products promote a constant overexpressed signal for cell cycle progression and division. The mutational profile of this cancer is copy number changes of genes and overexpression of selected gene products. For that reason, the methods developed here employ copy number values as the measurement for each node containing a gene in the signal transduction pathway and the resultant network that is employed to measure curvature.

This mutational profile of serous OC results in the loss of control for duplicating centrosomes, which sets up the polarity in a cell for the normal segregation of chromosomes. This is driven by the loss of function of p53 and the overexpression of cyclin E, which co-localizes with the centrosome, which duplicates abnormally producing three or more centrosomes^25^. In the extreme, this results in chromothripsis, where chromosome fragments and some of the parts are reassembled in a random order. This can result in double minute chromosomes without a centromere for proper segregation and random partition of the double minutes and distribution of multiple copy numbers. Often the population of cells forms a distribution of copy numbers of a combination of genes, which are then selected for optimal fitness.

Biomarkers of response to immunotherapy in OC remain underdeveloped. Here, we characterized a cohort of HGSOC patients treated with immunotherapy for whom detailed treatment, genomic, and survival data were available. Our analysis indicates that employing the copy number of the relevant genes as a measurement for each node in a network provides the strongest predictive power for OS, when compared to prior examined parameters such as TMB, LST, and FGA (Fig. 1). These results suggest that no one gene or even its alterations can predict responses to therapy. Rather it is the integration of the copy numbers of driver genes and the change of resultant networks formed by these genetic or epigenetic alterations that impacts immunological responsiveness of the tumor after checkpoint therapy. Employing the overexpression of the same set of genes and loss of p53 function in a mouse model of ovarian cancers treated with immunotherapy resulted in similar heterogeneous responses to checkpoint therapy and the beginnings of experimental tests of genes and products that could modify the results of the responses to cancer therapies^14^. This permits the pairing and testing of the type of modeling presented here along with prediction of genes with high curvature with experimental tests in a mouse model to improve the choice of therapies depending upon the genotypes of the tumors.

Interestingly, in non-small cell lung cancer a major tumor antigen, not genetically altered in sequence (not a neo-antigen), was found to be overexpressed in many different independent tumors^7,8^. This suggests that in serous OCs, like non-small cell lung cancers, the higher concentration of a non-genetically altered tumor antigen was an important variable in responsiveness to checkpoint therapy. Similar conclusions were reached by the mathematical construct employed here, measured by both abundance and changes in a network architecture and quantitated by curvature of the edges of the network.

For additional validation of our methodology, we tested our method on a much larger data set from Metabric (breast cancer) with 1903 samples. The KM plot is shown in Supplementary Fig. 14. To recap, network curvature was used in this study to investigate survival specifically in those women with recurrent HGSOC treated with ICI. Unlike other cancers, HGSOC has not been shown to respond well to ICI and traditional biomarkers, such as TMB, have not been predictive in HGSOC. In addition, in HGSOC to date, PD-L1 expression has unfortunately not been found to be predictive of response to ICI^26,27^. While the presence of TILs is prognostic in HGSOC^28^ and other cancers, their predictive value for ICI response is questionable. In two published studies that have evaluated combination of ICI with PARP inhibitors in HGSOC, the presence of TILs was not predictive of response^29,30^. Thus, while these biomarkers have been predictive of response to ICI in other cancer types, their value in HGSOC is rather limited. The demonstration of the predictive value of PD-L1 expression and TILs in the cohort analyzed in the current work would have been useful for comparison, but unfortunately, sufficient tissue to conduct such studies was not available.

Identification of novel biomarkers predictive of immunotherapy response in HGSOC is thus a high priority. The current study capitalizes on the unique biology of HGSOC (i.e., CNAs) and identifies curvature as a potential biomarker tool that can serve as a predictor of clinical benefit in patients treated with ICI. Notably, we feel that this tool is not simply prognostic, as our study investigated curvature as a predictor of OS in those with HGSOC not treated with ICI, and it was not significantly associated with prognosis, suggesting this is a biomarker truly related to ICI rather than HGSOC.

The marriage of mathematical models with experimental tests is one of the goals that will speed up the testing of new ideas and directions. The gene lists in Table 1 and Supplementary Tables 1–3 that compare the values of curvature, topology, geometry, feedback connectivity, and other properties of the networks under study, permit a selection of the best ways to measure lists of genes that impact success of immunotherapy. The conclusion of the analysis presented in this work is that the stability or instability of local network robustness driving changes in feedback connectivity has the largest impact upon prognosis after immunotherapy. The analysis identifies the mutant *TP53* gene and its loss of functional protein, resulting in the inability to control cyclin E activity and the resultant abnormalities in copying centrosome numbers accurately as the driving force for this cancer^23,25^.

In conclusion, a network version of the geometric concept of curvature was introduced to model information variability, robustness, and dysregulation of cancer gene networks. Total curvature, thus formulated for HGSOC, was demonstrated to work better in comparison to other standard metrics for the prediction of response to immunotherapy. Network curvature, formulated in this manner as a consistent information passing measure, thus appears to effectively capture global gene signaling dysregulation, and furthermore functions to identify key contributors to signaling dysregulation. Establishing total curvature as a useful clinical biomarker, possibly in combination with FGA (also proposed as a potential biomarker in ovarian cancer^12^), will require larger datasets in order to further quantify and validate these results.

## Methods

### Curvature background

We start with a brief, informal discussion on curvature to build some intuition before introducing the formal description of curvature as it was used in this work. See Supplementary Fig. 1 for an illustration of the key concepts.

Perhaps the most intuitive notion of curvature is that of *Gaussian curvature* on a surface^31^. The curvature proposed by Ollivier^6^ is the discrete analog of Gaussian curvature on a surface, and more generally, of Ricci curvature on higher dimensional objects. Application of this generalized, abstracted notion of curvature is proposed for studying cancer networks, as elucidated below. The key point is that the notion of curvature we employ is intrinsic to the given geometric object. For networks defined by graphs, one looks at such an intrinsically defined quantity to inform on its (functional) structure.

In the classical case, the Gaussian curvature of a surface is independent of how the surface is embedded in 3-dimensional space. Thus rather than look at the surface as it is embedded in 3-dimensional space from the perspective of an outsider, the key is to treat the surface as the space itself. With this approach, we can determine if the space is curved through the use of *geodesics*, the curves of (locally) shortest length between two points. (Geodesics generalize straight lines in Euclidean space.) One way to tell if the space is curved is to sum up the interior angles of a geodesic triangle. Geodesic triangles on a surface with positive (resp., negative) Gaussian curvature are *fat* (resp., *skinny*) compared to the triangle in Euclidean space. Loosely speaking, curvature can be inferred by the local behavior of geodesics—geodesics converge in regions of positive curvature and diverge in regions of negative curvature. On Riemannian manifolds, Ricci curvature is intimately related to the spread of geodesics emanating from the same point^31^.

While there are many ways to characterize the local behavior of Ricci curvature, we focus on Ollivier’s characterization that is relevant for our purposes: namely that in regions of positive (resp., negative) Ricci curvature, geodesic balls (on average) are closer (resp., farther) than their centers^31^. (A “geodesic ball” of radius *ϵ* centered at a given point *p* is defined as the image under the exponential map of the ball of radius *ϵ* on the tangent space at *p*). This is in contrast to Euclidean space where the distances between geodesic balls and their centers are the same. Ollivier’s characterization generalizes this notion of Ricci curvature applicable to graphs by replacing the geodesic balls with probability measures *μ*_*j*_^6^. In the Euclidean case, one may think of this as replacing points (delta functions) by small Gaussian balls (“fuzzified points”). The transportation distance between measures *μ*_*j*_ and *μ*_*k*_, prescribed by the Wasserstein distance *W*_1_, is used in lieu of the average distance between geodesic balls. The Wasserstein distance accounts for the geometry of the space and the distance between distributions associated with two nodes is related to the overlap of their neighborhoods. The rigorous mathematical details will be given now.

### Wasserstein distance

The Wasserstein distance is a particular instance of the OMT problem. It is a natural candidate for comparing probability measures because it accounts for both the shape of the distributions (i.e., weighted values) and the distance on the underlying space. The OMT problem, originated by Gaspard Monge^32^, seeks the optimal way to redistribute mass with minimal transportation cost. Leonid Kantorovich reformulated and relaxed the problem in the context of resource allocation^33^; for more details, see^34–36^. We consider the following discrete formulation. Since we will be applying the theory to weighted graphs, this will be sufficient.

Accordingly, let ${{{\mathcal{X}}}}$ denote a metric measure space equipped with distance *d*(⋅,⋅). Given two (discrete) probability measures *μ*_0_ and *μ*_1_ on ${{{\mathcal{X}}}}$, the *Wasserstein distance* *W*_1_ between *μ*_0_ and *μ*_1_ is defined as

1 $${W}_{1}({\mu }_{0},{\mu }_{1}):= \mathop{\inf }\limits_{\pi \in {{\Pi }}({\mu }_{0},{\mu }_{1})}\mathop{\sum}\limits_{x,y}{\pi }_{xy}d(x,y),$$

where Π(*μ*_0_, *μ*_1_) is the set of joint probabilities on ${{{\mathcal{X}}}}\times {{{\mathcal{X}}}}$ with marginals *μ*_0_ and *μ*_1_. Here, *π*_*xy*_ may be interpreted as the amount of mass moved from *x* to *y* and the cost of transporting a unit of mass is taken to be the distance traveled (i.e., *d*). Thus, the Wasserstein distance (1) gives the minimal net cost of transporting mass distributed by *μ*_0_ to match the distribution of *μ*_1_. The OMT problem therefore seeks the optimal *transference plan π* ∈ Π(*μ*_0_, *μ*_1_) found to be the infimal argument for which the Wasserstein distance is realized.

As is well-known, the computation of *W*_1_ may be reduced to one of linear programming^34^. One can consider a dual version of the problem based on work Beckmann^37^, which reduces the computation to one of optimizing over a certain set of fluxes, upon which we based our code. Details may be found in^38^.

### Curvature

The interplay between OR curvature, network entropy, and functional robustness is linked by OMT and is rich in theory. We outline this now, beginning with the OR curvature^6^.

Based on the work of von Renesse and Sturm^16^, Ollivier extended the notion of Ricci curvature, defined on a Riemannian manifold, to discrete metric measure spaces^6^. Specifically, let ${{{\mathcal{X}}}}$ be a metric measure space equipped with a distance *d* such that for each $x\in {{{\mathcal{X}}}}$, one is given a probability measure *μ*_*x*_. The probability measure *μ*_*x*_ can be thought of as *fuzzifying* or *blurring* the point *x*. For two points $x,y\in {{{\mathcal{X}}}}$, OR curvature is defined as

2 $${\kappa }_{\rm{OR}}(x,y):= 1-\frac{{W}_{1}({\mu }_{x},{\mu }_{y})}{d(x,y)},$$

where *W*_1_ is the *Wasserstein distance*.

### Curvature on graphs

For our purposes, the metric measure space is taken to be a weighted graph *G* = (*V*, *E*) with nodes (vertices) *V* and edges *E*. *G* is assumed to be a *simple, connected* and *undirected* graph. Instead of points *x* in a metric space, we now consider nodes *x*_*j*_ ∈ *V*, denoted simply by its subscript *j*. In this work, the graph is constructed as follows. Each node *j* ∈ *V* represents a gene; hereafter node and gene are used interchangeably. Edges *e* = (*j*, *k*) ∈ *E* define known interactions between genes (nodes) at the protein level (here given by HPRD) and *j* ~ *k* denotes that *k* is a neighbor of *j*. We then incorporate copy number (CN) values as nodal weights, denoted *w*_*j*_. Note that for *j* ∈ *V*, we take *w*_*j*_ = (*CN*)_*j*_ + 1; the affine translation is used to ensure all weights are positive.

We treat the weighted graph as a Markov chain. In this context, the probability measure *μ*_*j*_ attached to node *j* ∈ *V* can be thought of as the probability of a 1-step random walk starting from node *j*. The 1-step transition probability *p*_*jk*_ of going from *j* to *k* is expressed by the *principle of mass action*^39^. According to this principle, if there is a known connection between gene *j* and gene *k* (i.e., (*j*, *k*) ∈ *E*), then the probability that they interact is proportional to the product of their CN values:

3 $${p}_{jk}\propto {w}_{j}{w}_{k}.$$

Normalizing the mass action over all possible edges to ensure that *p*_*jk*_ is a probability, i.e., ∑_*j*~*k*_*p*_*jk*_ = 1, we define the transition probabilities *p*_*jk*_ of the stochastic matrix *P* = [*p*_*ij*_] associated with the Markov chain as follows:

4 $${p}_{jk}=\left\{\begin{array}{ll}\frac{{w}_{k}}{{\sum }_{j \sim l}{w}_{l}},\quad &{{{\rm{if}}}}\ j \sim k\\ 0,\quad &{{{\rm{otherwise}}}}.\end{array}\right.$$

Accordingly, for each gene *j*, we associate a probability measure *μ*_*j*_ defined on the node set *V* with *n* associated nodes

5 $${\mu }_{j}=[{p}_{j1},{p}_{j2},...,{p}_{jn}],\quad j=1,...,n.$$

Alternatively, *μ*_*j*_ can be thought of as *fuzzifying* the node *j* over its 1-step neighborhood.

#### Graph distance

We have now specified the points (*x*) and measures (*μ*_*x*_) needed to compute OR curvature in Eq. (2) on a graph. All that remains is the distance *d*(*x*, *y*). In lieu of the commonly used *hop distance*, i.e., the distance between two nodes *j*, *k* ∈ *V* that is defined as the shortest path length over all paths connecting *j* and *k*, we take the corresponding graph distance *d*_*jk*_ to be the *weighted hop distance* (whop).

More precisely, for fixed nodes *j* and *k*, let *P*^*jk*^ denote a path connecting them. Let $\{{w}_{1}^{jk},\ldots ,{w}_{n}^{jk}\}$ be the set of all the associated edge weights. Then we set

6 $$\ell ({P}^{jk}):= \mathop{\sum }\limits_{i=1}^{n}\frac{1}{{w}_{i}^{jk}}.$$

Denoting by ${{{\mathcal{P}}}}:= \{{P}_{1}^{jk},\ldots ,{P}_{m}^{jk}\},$ the set of all possible paths connecting *j* and *k*, we define the *weighted hop distance (whop)* between *j* and *k* to be:

7 $${d}_{jk}:= \mathop{\min }\limits_{1\le u\le m}\ell ({P}_{u}^{jk}).$$

Note that the edge weights *w*_*uv*_ for all edges *e* = (*u*, *v*) ∈ *E* are constructed as

8 $${w}_{uv}:= \frac{{p}_{uv}+{p}_{vu}}{2}.$$

This formulation was chosen so the distance between two nodes is inversely related to the probability of their interaction. Thus, the higher (resp., lower) the probability of two nodes interacting, the smaller (resp., larger) the distance between them should be. The average is taken merely so the distance is symmetric, i.e., *d*_*jk*_ = *d*_*kj*_. See Supplementary Figs. 2 and 3 for an explicit example of the weighted hop distance on a simple network.

#### Edge curvature

With the choice of graph distance in Eq. (7), the OR curvature in Eq. (2) can now be computed between any two nodes in the graph. Due to the large nature of the graphs of interest, we constrain the curvature computation to edges. Notice, from the curvature definition in Eq. (2), the ratio $\frac{{W}_{1}({\mu }_{j},{\mu }_{k})}{{d}_{jk}}$ relates the transport cost of moving the distribution (i.e., *fuzzy* ball) associated with *j* to *k* to the ground distance. Informally, the more the neighborhoods of two nodes overlap, the lower the transportation cost between them and thus the higher the curvature associated with the edge. As such, curvature informs on the local functional relationship between neighborhoods.

#### Scalar and total curvature on graphs

In order to obtain a node-level measure of curvature, we consider a contraction of the edge curvatures, analogous to *scalar curvature* defined on points of a manifold in Riemannian geometry^31^. Motivated by the notion of *signaling entropy rate* in information theory^40^, we define the (nodal) *scalar curvature* of gene *j* to be the weighted sum of the curvatures on all edges incident to *j*:

9 $${\kappa }_{j}:= {\pi }_{j}\mathop{\sum }\limits_{j \sim k} {\kappa }_{\rm{OR}}(j,k),$$

where the weight *π*_*j*_ is the *j*th component of the stationary distribution *π* associated with the Markov chain *P*:^39^

10 $$\pi =\pi P,\quad \mathop{\sum}\limits_{j}{\pi }_{j}=1.$$

The stationary distribution in this setting (connected graph) is also the limiting distribution of the Markov chain, known as the *stationary* or *equilibrium* distribution. Thus, the quantity *π*_*j*_ describes the relative importance of node *j* with respect to all other nodes. We therefore scale the nodal curvature by its component in the stationary distribution in order to correct for nodal bias. Furthermore, the stationary distribution has a closed form that may be easily computed as follows:

11 $${\pi }_{j}=\frac{1}{Z}{w}_{j}\mathop{\sum}\limits_{j \sim k}{w}_{k}$$

where *Z* is the normalization factor. We note that unweighted and alternative weightings have been proposed^38,41^.

Lastly, we define the *total curvature* *κ*_*G*_ of a network to be the net scalar curvature, summed over all nodes in the graph

12 $${\kappa }_{G}:= \mathop{\sum}\limits_{j}{\kappa }_{j}.$$

### Curvature and robustness

One of the main motivations for using curvature to study networks in general, and biological networks in particular, is its theoretical connection to network robustness. Given its importance, we outline the argument here which also gives a justification for using OR curvature^6^.

We begin by noting that Sturm^16^, Lott and Villani^15^ related a lower bound on the Ricci curvature of a smooth Riemannian manifold to the entropy of densities along a constant-speed geodesic with the use of the Wasserstein distance. This laid the groundwork for the connection between curvature, entropy, and the Wasserstein metric, and led to the remarkable observation that changes in Ricci curvature Δ*κ*_Ric_ are positively correlated with changes in (Boltzmann) entropy Δ*S*:

13 $${{\Delta }}{\kappa }_{\rm{Ric}}\times {{\Delta }}S\ge 0.$$

The positive correlation between changes in curvature Δ*κ*_Ric_ and changes in robustness Δ*R*:

14 $${{\Delta }}{\kappa }_{\rm{Ric}}\times {{\Delta }}R$$

is realized by Eq. (13) and the fluctuation theorem^42^ from large deviations theory indicates that changes in entropy are positively correlated with changes in robustness Δ*R*:

15 $${{\Delta }}S\times {{\Delta }}R.$$

Here, *robustness* refers to the ability of a system to recover or maintain its ability to function after it is perturbed in some way (e.g., stress signal). The OR curvature on networks is directly derived from the Lott–Sturm–Villani relationship, and thus was chosen over other possible discrete models^43–46^.

Curvature’s intimate connection to robustness makes it a particularly attractive method for analyzing key nodes and interactions in large, complex PPI networks. This connection is linked by entropy as shown in Eqs. (13) and (15), bridging this geometric analysis to an interesting perspective on the relationship between the topological and functional properties of the weighted network. With this notion of the change in curvature as a proxy for the more qualitative notion of functional robustness, we rank genes according to the change in curvature with respect to the topology and between sub-groups identified; see the following “Results” section.

### Data description and processing

In this section, we outline the data description and processing that we used in our HGSOC analysis. Further details about the data may be found in^12^.

First of all, TMB was calculated by dividing the number of non-synonymous mutations by the total size of the capture panel in megabases. Secondly, based on the CNAs by FACETS, FGA was defined as the cumulative length of segments with ${{\mathrm{log}}}\,2$ or linear CNA value larger than 0.2 divided by the cumulative length of all segments measured. LST scores, defined as a chromosomal breakpoint resulting in allelic imbalance between adjacent regions of at least 10Mb, were determined, and a cutoff ≥15 was employed for LST-high cases.

**Fig. 4:**
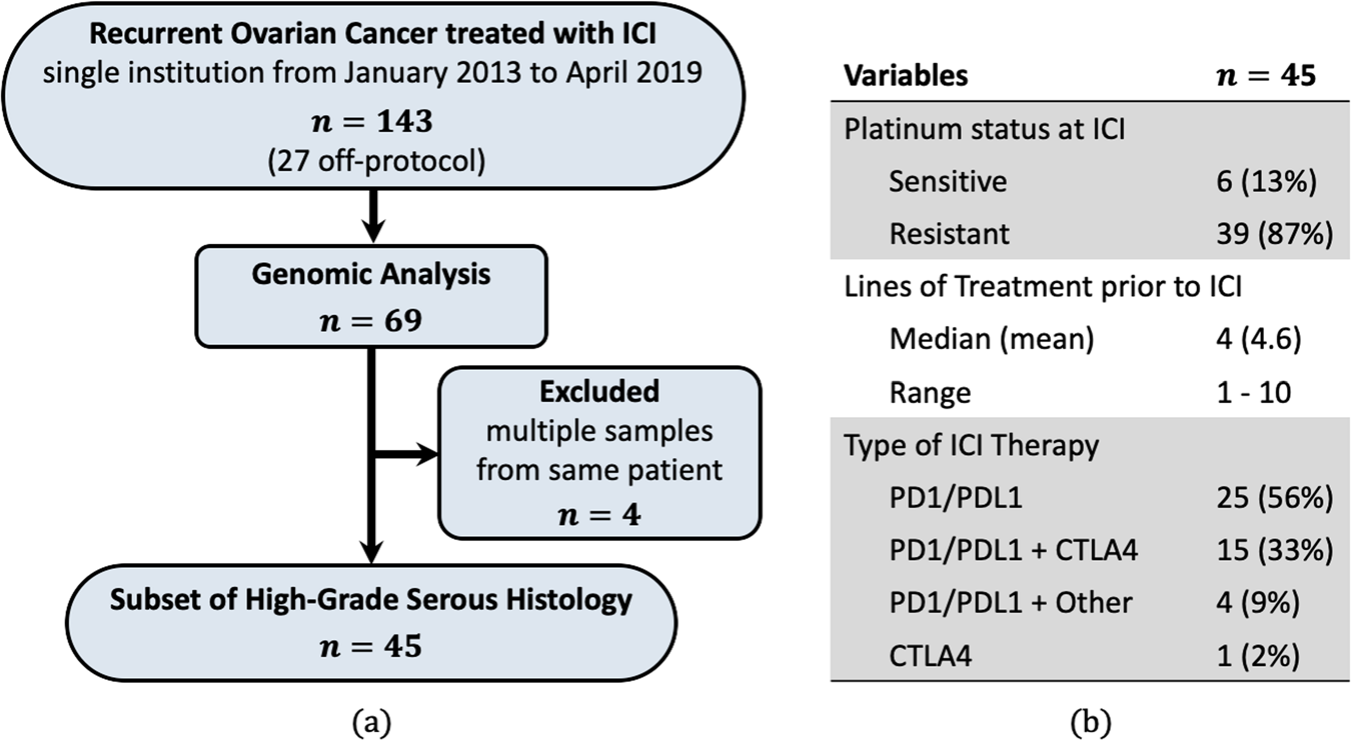
Patient selection and clinical characteristics. **a** Patient selection. **b** Clinical characteristics of patients with recurrent HGSOC administered ICI therapy.

Next, regarding the data characteristics, we used DNA gene CNA data from a subset of 69 women with recurrent OC who received immunotherapy from a previously published series^12^. The subtypes of ovarian cancer are in fact quite different diseases, originating in different cell types and being caused by distinct mutations with diverse outcomes, and should therefore be analyzed separately^19^. Accordingly, we restrict our re-analysis to a subset of samples (*n* = 49) with HGSOC, which is the most common and lethal subtype. Four HGSOC patients had two samples, and the replicate samples were removed from the analysis. This resulted in a total of 45 tumor samples, 32 of which were metastases and 13 represented primary (adnexal) tumors, with 22 and 10 deaths in each group, respectively, at the time the study group was analyzed. This forms a homogeneous group of cancers (Fig. 4). Tumor and normal samples from the 45 patients were profiled utilizing the FDA-cleared Memorial Sloan Kettering Integrated Mutation Profiling of Actionable Cancer Targets (MSK-IMPACT) sequencing assay, their mean age was 58 years, and mean TMB was 5.9. Patient selection and clinical characteristics are displayed in Fig. 4 and in Table 2.

**Table 2:** HGSOC patient characteristics.

	All patients	Low curvature	High curvature	
Characteristic	(*n* = 45)	(*n* = 12)	(*n* = 33)	*p*
Age at diagnosis (years)				0.062
Mean ± SD	58.0 ± 9.3	62.3 ± 7.5	56.4 ± 9.4	
Range	27.0–75.0	49.0–75.0	27.0–75.0	
Median (IQR)	58.0 (52.0–64.0)	64.0 (58.8–65.5)	55.0 (51.0–61.0)	
Age at start of ICI (years)				0.023
Mean ± SD	62.1 ± 8.7	67.1 ± 6.9	60.3 ± 8.7	
Range	37.0–78.0	55.0–78.0	37.0–77.0	
Median (IQR)	62.0 (56.0–69.0)	67.0 (63.3–70.3)	59.0 (55.0–66.0)	
Stage at diagnosis				0.502
III	25	8	17	
IV	20	4	16	
Time from diagnosis to start of ICI (months)				0.581
Mean ± SD	50.9 ± 35.9	58.8 ± 43.1	48.1 ± 33.2	
Range	5.3–166.0	17.4–166.0	5.3–123.1	
Median (IQR)	49.4 (23.3–61.7)	44.5 (33.4–61.8)	49.4 (18.5–61.7)	
Duration of ICI (weeks)				0.807
Mean ± SD	20.2 ± 23.6	14.3 ± 8.6	22.3 ± 27.0	
Range	0.1–143.0	0.7–28.3	0.1–143.0	
Median (IQR)	12.3 (7.7–23.1)	13.6 (7.8–20.3)	12.1 (7.7–23.1)	
Overall survival (months)				0.007
Mean ± SD	16.7 ± 11.7	8.8 ± 7.2	19.6 ± 11.7	
Range	0.4–44.8	0.4–27.4	0.4–44.8	
Median (IQR)	15.3 (6.5–24.6)	7.4 (4.9–10.9)	20.3 (11.1–26.0)	
Sample type				0.010
Metastasis	32.0	12	20	
Primary	13.0	0	13	
Status at last follow-up				0.134
Alive	13	1	12	
Dead	32	11	21	
TMB				0.959
Mean ± SD	3.7 ± 2.3	3.5 ± 1.9	3.8 ± 2.5	
Range	1.0–9.7	1.1–6.7	1.0–9.7	
Median (IQR)	3.3 (2.0–4.4)	2.6 (2.0–5.3)	3.3 (2.0–4.4)	
FGA				0.005
Mean ± SD	0.4 ± 0.2	0.3 ± 0.2	0.5 ± 0.2	
Range	0.005–0.871	0.005–0.629	0.092–0.871	
Median (IQR)	0.4 (0.3–0.6)	0.2 (0.1–0.4)	0.5 (0.4–0.6)	
LST				0.024
Mean ± SD	25.0 ± 10.3	19.3 ± 9.1	27.1 ± 10.0	
Range	2.0–51.0	2.0–32.0	2.0–51.0	
Median (IQR)	25.0 (20.0–29.0)	22.0 (13.5–25.8)	27.0 (22.0–34.0)	
Platinum status at ICI				1.000
Platinum Sensitive	6	1	5	
Platinum Resistant	39	11	28	
BRCA1/2 Status				1.000
Wild-type	35	9	26	
Mutation	10	3	7	
ICI target				0.909
PD-1/PD-L1	25	6	19	
PD-1/PD-L1 + CTLA-4	15	5	10	
PD-1/PD-L1 + other	4	1	3	
CTLA-4	1	0	1	

*P* values were obtained using two-sided Wilcoxon–Rank Sum test for continuous variables and Fisher-exact test for categorical variables.

*SD* standard deviation, *IQR* interquartile range.

CN segments were mapped to individual genes according to GRCh37 and for each sample, each gene was assigned the maximum CN value of all segments that mapped to it. After removing all genes with missing data and all genes not in the HPRD network, we extracted the set of genes comprising the largest connected network (Supplementary Fig. 11). This resulted in a CNA data matrix of size 3489 (genes) × 45 (samples).

The network topology was constructed as follows. Edges between genes were defined by the PPI obtained from HPRD^1,2^. The network topology was then taken to be the largest connected component in the HPRD network restricted to the set of genes in our data set. This resulted in a network with 9710 edges and 3489 nodes with an average degree of 5.57. The rationale is that the established interactions between gene products serve as a viable proxy for the functional connectivity at the gene level.

Subject-specific networks were created by assigning nodal weights *w*_*j*_ prescribed by the CN value. Specifically, the CN data took on discrete integer values in the range [0, 38]. In order to ensure all weights were positive, we used the translation *w*_*j*_ = *x*_*j*_ + 1 where *x*_*j*_ is the CN value for gene *j*. For each subject, Markov chains were computed as defined in Eq. (4) followed by the associated stationary distribution in Eq. (11). Next, OR curvature using Eq. (2) was computed on each edge in the fixed network, scalar curvature defined in Eq. (9) was subsequently computed for each node and lastly, total curvature using Eq. (12) was computed for the network. A critical aspect of the curvature analysis is that it provides a *relative* quantity and it is the *change* in curvature that is of interest, indicative of changes in the network’s capacity for communication. Thus, we would expect that patients whose samples have a lower total curvature (i.e., a relative net decrease in capacity) would be associated with a poorer prognosis than those with higher total curvature values.

### Ethics statement

All data were approved for analysis (MSK IRB protocol #15-200), including an institutional tissue banking protocol (#06-107) and molecular profiling protocol (#12-245), with all study participants providing written informed consent to participate.

### Reporting summary

Further information on research design is available in the Nature Research Reporting Summary linked to this article.

## Supplementary information

Supplementary Information

Reporting Summary

## Data Availability

The data analyzed in this study are publicly available from cBioPortal at https://www.cbioportal.org/study/summary?id=hgsoc_msk_2021.

## Code availability

The code written in Python (version 3.9) is available upon reasonable request.
