# Supplementary material for: Geometric network analysis provides prognostic information in patients with high grade serous carcinoma of the ovary treated with immune checkpoint inhibitors
Source: NPJ Genom Med. 2021 Nov 24;6:99. doi: 10.1038/s41525-021-00259-9 (PMC8613272; doi:10.1038/s41525-021-00259-9)
Supplement: Supplementary file 1 — Supplementary Information [file 41525_2021_259_MOESM1_ESM.pdf]

Supplementary material

Background on curvature

The relationship between the continuous and discrete notions of curvature are summarized in Supplementary Figure 1.

Weighted hop distance

Using Zachary's Karate Club graph [44] as an example, the resulting whop distance for all edges is shown in Supplementary Figure 2. A more detailed comparison between the hop and whop distances, illustrated by heat maps of the corresponding distance matrices of all node pairs in the network, is shown in Supplementary Figure 3.

Validation of survival analysis in HGSOC

In this work, curvature is used as a relative measure of network response to immunotherapy in HGSOC for predicting survival. An independent data set for HGSOC patients treated with immunotherapy was not available for external validation and the sample size was too small to separate into training and validation sets. We therefore performed three tests for internal validation. K-fold cross-validation (K-CV) [45] (Supplementary Figure 4) and bootstrap validation (Supplementary Figure 5) were performed to test the validation. In addition, to test if the significant difference in survival found between the high and low curvature groups would likely be observed regardless of the initial gene level data, the CN data was randomly permuted and reassigned amongst the genes. The curvature pipeline was subsequently reperformed with the randomized node weightings and the survival between the two groups defined by low and high curvature according to the 25th percentile of the total curvature value was reassessed in the same manner using the log-rank test. This random process was repeated 500 times with zero out of the 500 trials resulting in a log-rank test statistic greater than the unpermuted sample based test statistic, suggesting that the null hypothesis may fairly be rejected and that total curvature is statistically likely to be picking up on real signals of functional robustness providing a relative measure of overall survival in response to immunotherapy.

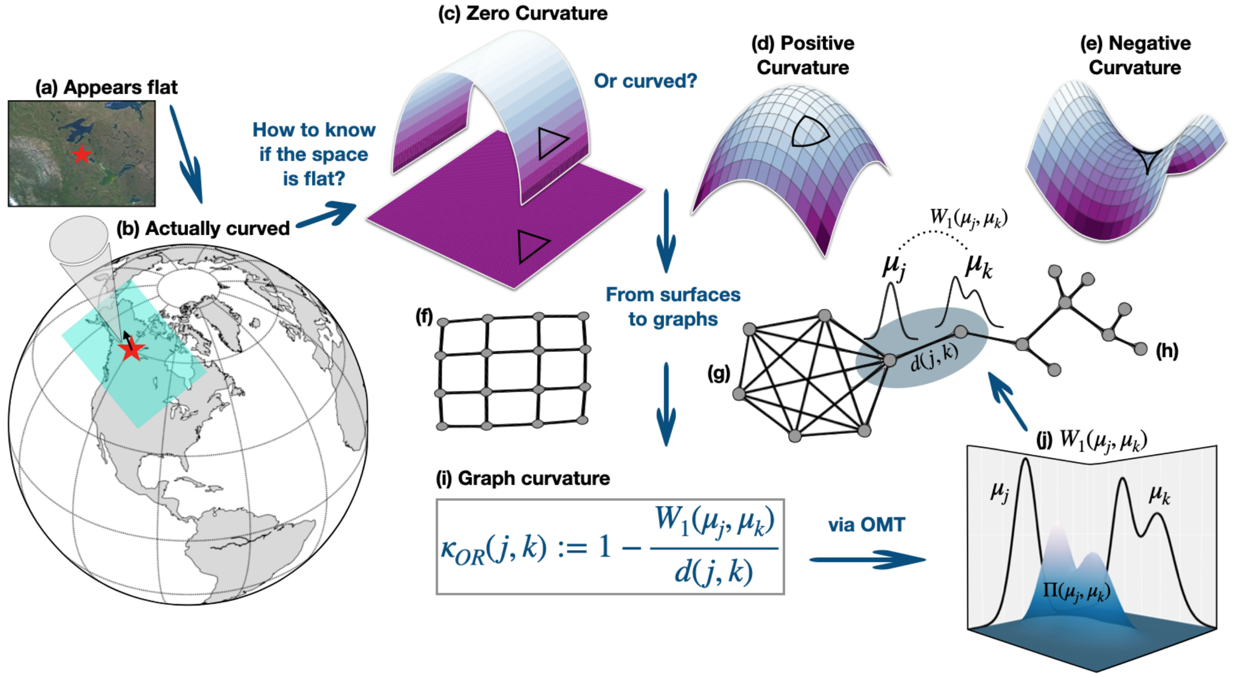

Supplementary Figure 1: **Curvature intuition on graphs.** Curvature is an intrinsic property of a surface, and therefore does not depend on how it is situated in space. For example, (b) we know the Earth is curved, (a) even though it appears flat when standing on its surface. Similarly, (c) a plane that is bent into an arc still has zero curvature. The apparent curvature is merely due to how it is embedded in space. Examples of canonical surfaces with zero, positive and negative curvature are shown, respectively, in (c), (d) and (e). Geodesic triangles can be used to determine the curvature of a surface without specifying its embedding. Compared to (Euclidean) flat space (c), fat (d) and skinny (e) triangles are characteristic of positive and negative curved spaces, respectively. Going from smooth surfaces to graphs, (f) a grid is analogous to a surface with zero curvature while (g) many triangles (indicative of redundancies or feedback mechanisms) are characteristic of graphs with positive curvature and (h) tree-like topologies (indicative of diverging paths) are characteristic of graphs with negative curvature. (i) On a graph, curvature between two nodes j and k is characterized by the ratio of the transport distance $W_1(\mu_j, \mu_k)$ between distributions μ_j and μ_k (defined respectively on nodes j and k) and the underlying ground distance $d(j, k)$ between the two nodes. The transport distance (W_1) comes from the theory of optimal mass transport (OMT) and provides a *functional* distance between the nodes that accounts for the shape of the distribution and amount of shared neighbors. Curvature is positive (resp., negative) when the transport distance (i.e., information) between nodes is smaller (resp., larger) than the ground distance between them, reflecting the ease with which information is shared between nodes.

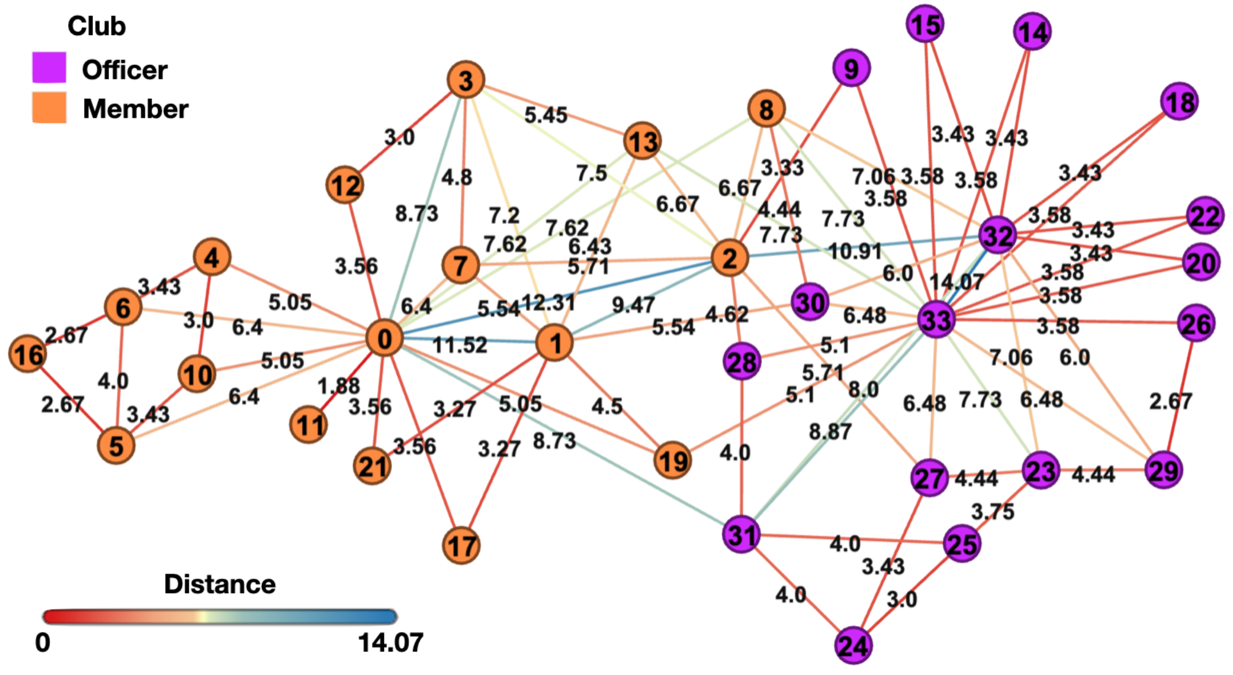

Supplementary Figure 2: **Weighted hop distance.** The weighted hop distances are shown for every edge in Zachary's Karate Club Graph [44] with all node weight values initialized equal to 1. The node color indicates if the corresponding person is a club officer (purple) or member (orange). The distance between edge-adjacent nodes is shown at the edge midpoint.

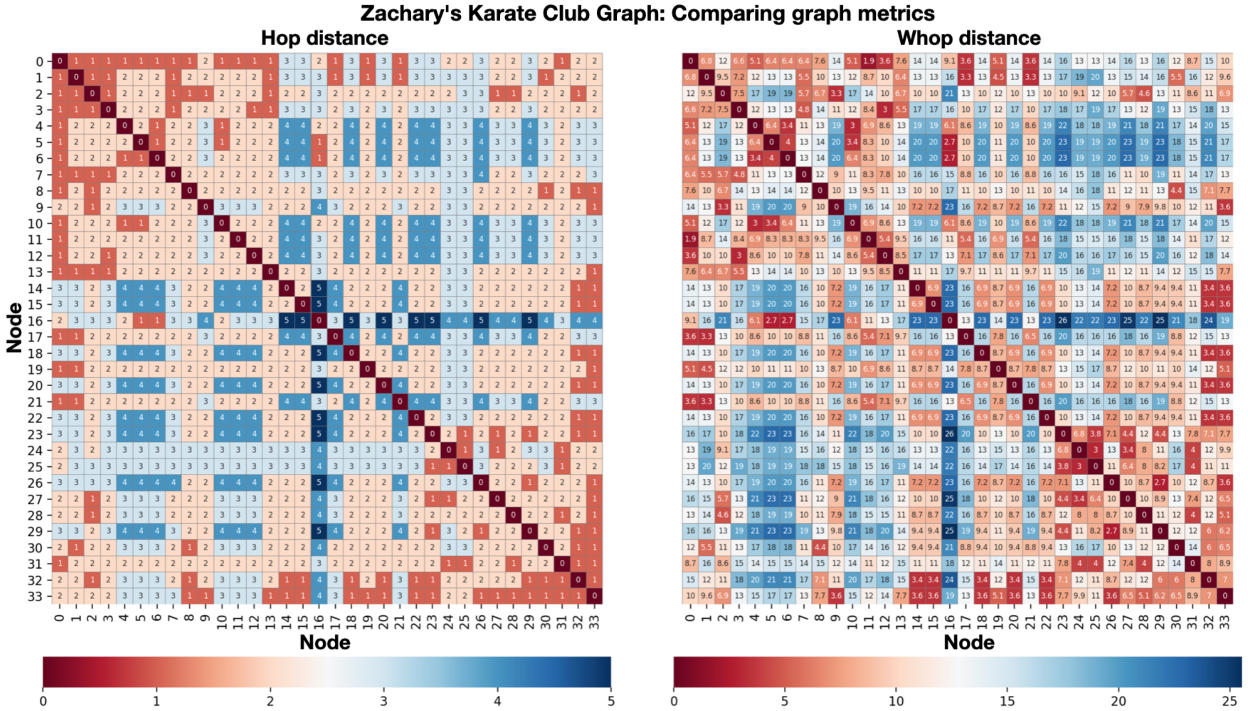

Supplementary Figure 3: **Comparing graph metrics.** The distances between every two nodes in Zachary's Karate Club Graph [44], with all node weight values initialized equal to 1, are shown using (left) the hop distance and (right) weighted hop distance.

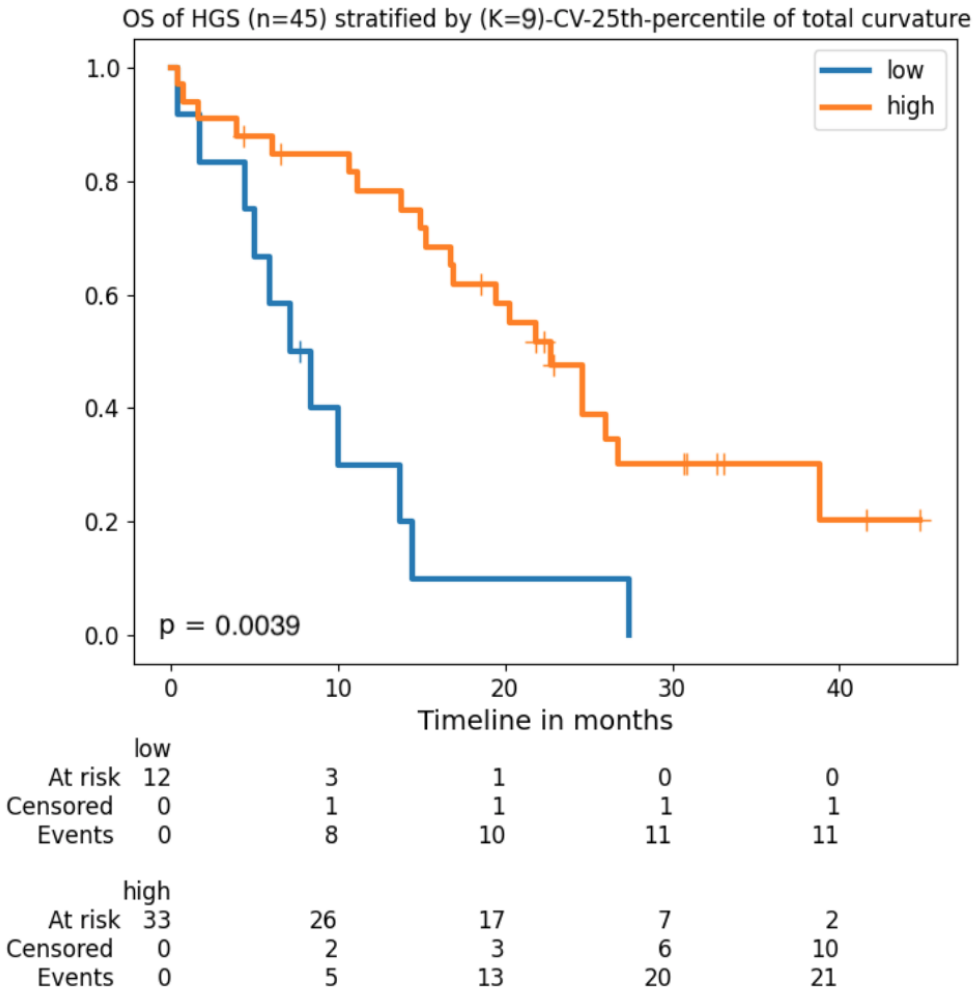

Supplementary Figure 4: ***K*-fold cross-validation.** For HGS (n=45), 9-fold cross validated classification Kaplan-Meier curves and p-value determined from the empirical null distribution of the cross-validated log-rank statistic [45]. Briefly, the K-CV was performed as follows: the data set was partitioned into K groups of size n/K where n is the total number of samples (here, $n = 45$, $K = 9$ and $n/K = 5$). The n/K samples in the first partition were removed from the set. The cut for classification was determined to be the 25th percentile of the total curvature from the remaining $n - n/K$ samples. Each of the n/K removed samples was then classified as *low* or *high* curvature by comparing their total curvature to the cutoff. The n/K samples were then returned to the set and the process was repeated for each partition. This process terminated with each sample being classified by $n - n/K$ other samples resulting in two cross-validated high and low curvature groups. KM survival analysis was then performed between the predicted high and low risk groups and a log-rank statistic was computed. The 9-fold CV procedure was repeated 10,000 times. Out of the 10,000 tests, 39 trials resulted in cross-validated log-rank statistic greater than that of original data with test statistic = 9.499, resulting in a significance level of 0.0039.

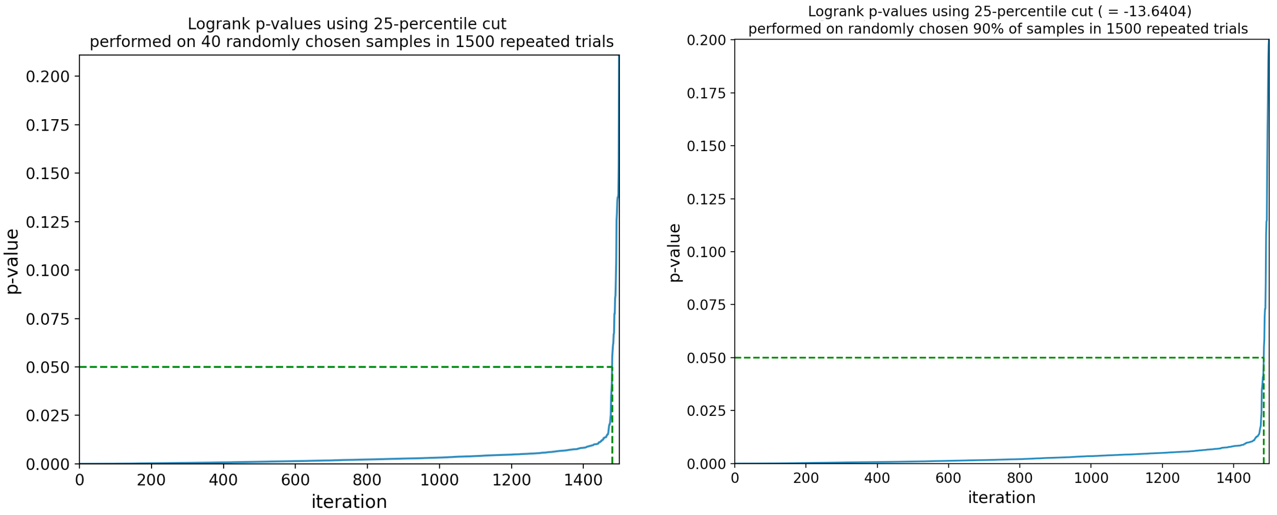

(a) bootstrap cutoff

(b) fixed cutoff

Supplementary Figure 5: **Bootstrap validation.** HGSOC (n=45) bootstrapped validation for Kaplan-Meier survival analysis. Bootstrap samples were generated by randomly selecting approximately 90% ($n = 40$) for the HGSOC. The bootstrap sample was then stratified into high and low curvature groups by either (a) the bootstrap cutoff (the 25th percentile of the bootstrapped set's total curvature values) or (b) the fixed cutoff (the 25th percentile of the original set's total curvature values). In both cases, survival of each bootstrap group was estimated by the Kaplan-Meier analysis and the log-rank p-value was used to test if the survival curves for each group were different. P-values resulting from 1,500 iterations of this process are shown in increasing order by the blue lines. The dotted green lines emphasize the significance level at $\alpha = 0.05$. Out of the 1,500 trials, (a) 20 bootstrap-cutoff trials resulted in $p > 0.05$ and (b) 15 fixed-cutoff trials resulted in $p > 0.05$.

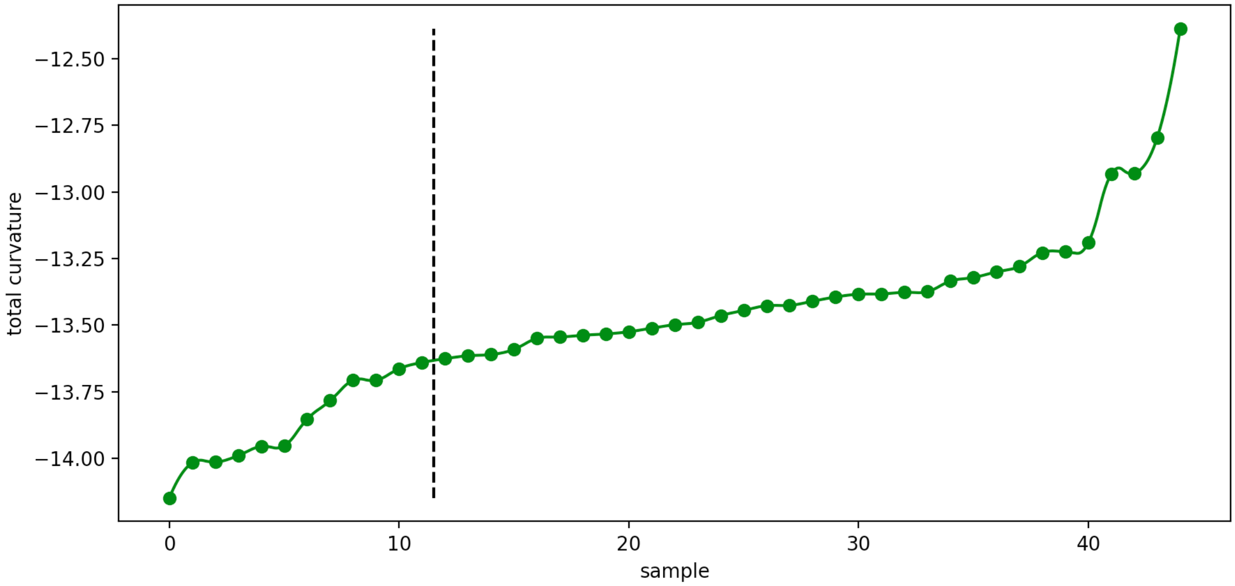

Supplementary Figure 6: **Rationale for selecting 25th-percentile cutpoint.** The sorted total curvatures of all samples are shown by green circles. The dotted black line separates the low and high classified samples, where curvature is seen to start increasing slowly by the fitted line.

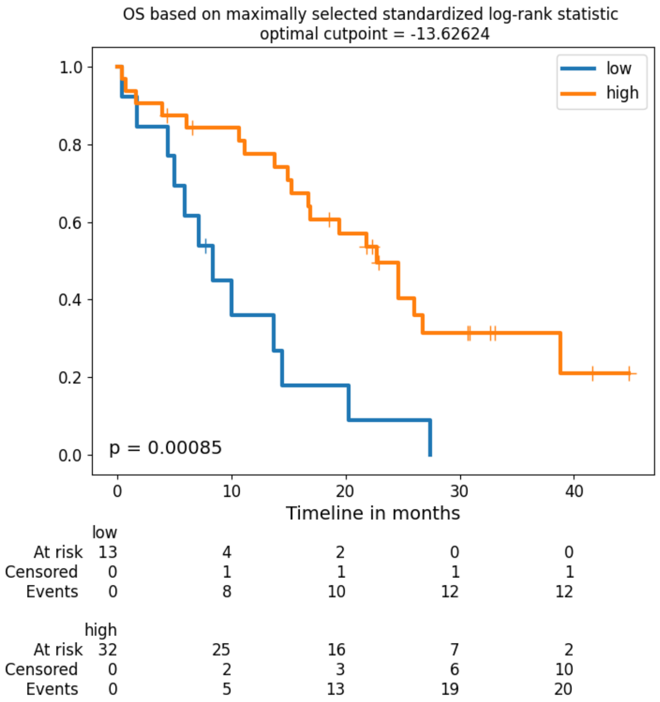

Supplementary Figure 7: **Alternative cutpoint selection.** Kaplan-Meier survival analysis using alternative cutpoint for identifying high and low curvature groups with the maximally selected log-rank statistic [20, 21] (cutpoint = -13.62624, requiring 25% minimal proportion) using R's MAXSTAT package. Note that this results in one sample being moved from the high curvature to the low curvature group as found by the 25th percentile of total curvature cutoff.

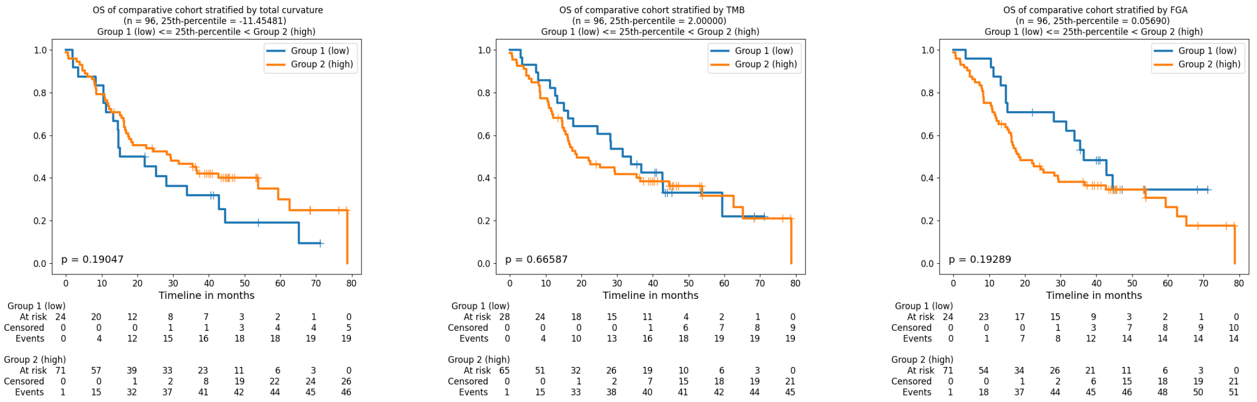

(a) total curvature (b) TMB (c) FGA

Supplementary Figure 8: **Survival curves for HGSOC samples that did not receive ICIs.** HGSOC samples (IMPACT data, $n = 96$) stratified into low and high groups by the 25th percentile of total curvature and available genomic parameters. Overall survival is defined as the duration from the time of diagnosis to death or time of last follow-up and p-values were derived from the log-rank test.

Survival analysis on metastasis cohort

Kaplan-Meier survival analysis as performed in Figure 1 was repeated on the metastasis cohort ($n = 32$). The corresponding survival curves and log-rank p-values are shown in Supplementary Figure 9.

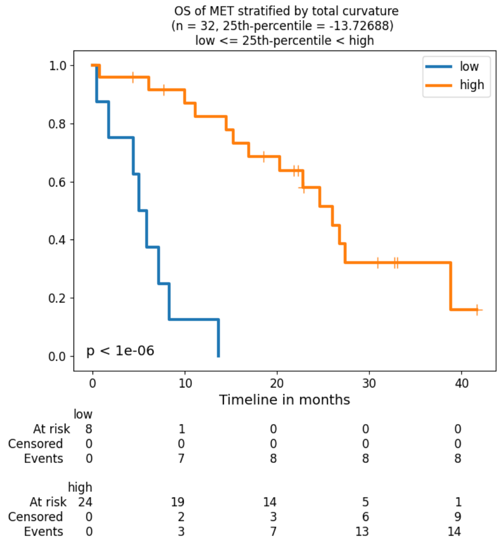

(a) total curvature

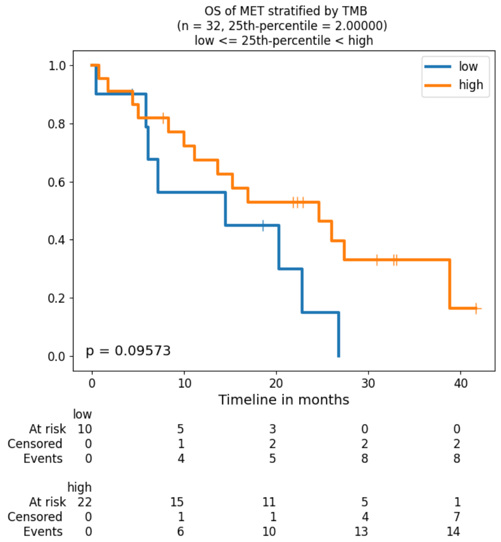

(b) TMB

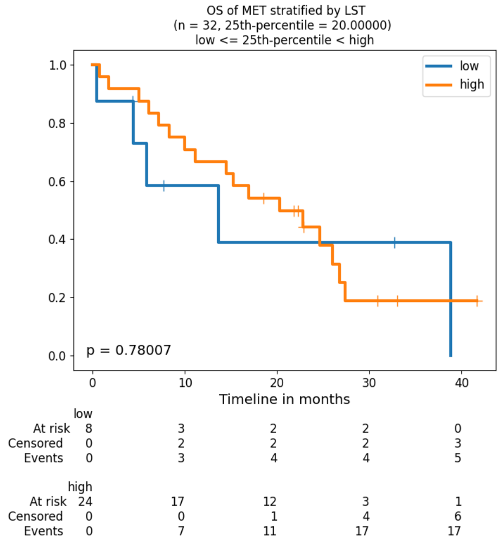

(c) LST

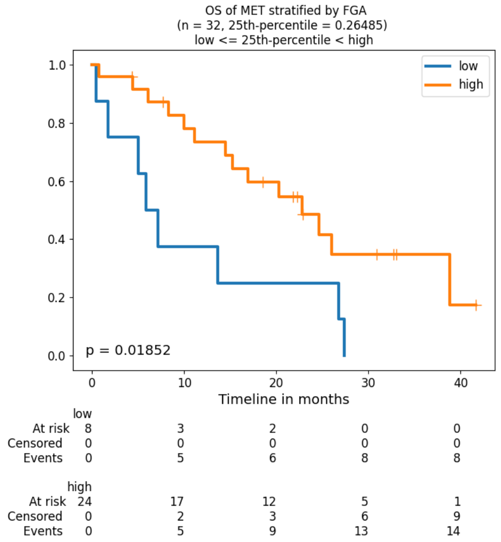

(d) FGA

Supplementary Figure 9: **Survival curves for HGSOC metastasis samples** ($n = 32$)**.** The samples were stratified into low and high groups by the 25th percentile of total curvature and genomic parameters. P-values were derived from the log-rank test.

Top curvature ranked genes

In this section, we list in Supplementary Tables 1, 2, 3 the top ranked genes according to the criteria described in Section 2.2. We list both the greatest positive and negative curvature differences. Finally, in Supplementary Table 4, we list (in alphabetical order) the key genes found by the curvature analysis using all the comparisons, and then in Supplementary Table 5, we give the 100 identified candidate genes based on risk listed alphabetically.

Supplementary Table 1: Changes in average scalar curvature based on overall survival (OS)

Rank	Gene	$\Delta\kappa_{OS} > 0$	Gene	$\Delta\kappa_{OS} < 0$
0	TP53	0.059206	CTNNB1	-0.029402
1	SMAD3	0.055706	CREBBP	-0.026285
2	ATXN1	0.028886	MYC	-0.024026
3	EP300	0.025355	PTK2	-0.023118
4	TGFBR1	0.017961	AR	-0.021899
5	AKT1	0.015810	SHC1	-0.018774
6	JUN	0.015342	SMAD2	-0.015914
7	SRC	0.013336	RB1	-0.015029
8	ACTB	0.013162	VIM	-0.011736
9	PCNA	0.010664	PRKCA	-0.010209
10	ESR1	0.010068	SMAD4	-0.009694
11	CDKN1A	0.009641	MAPK1	-0.008352
12	RAC1	0.009197	GRB2	-0.008095
13	CDKN1B	0.008439	SVIL	-0.005490
14	PRKCD	0.008138	APP	-0.005477
15	HSP90AA1	0.007070	SMARCA4	-0.005419
16	CCNE1	0.005931	PARP1	-0.005321
17	STAT1	0.005750	FN1	-0.005122
18	COPS6	0.005661	PIK3R2	-0.004975
19	MAPK14	0.005247	CRMP1	-0.004917
20	MDFI	0.005216	MAPK8	-0.004334
21	SMURF1	0.005144	ITGB1	-0.004226
22	CDK5	0.004177	HTT	-0.004059
23	ACTN1	0.004095	HSF1	-0.004010
24	YWHAE	0.003982	INSR	-0.003442
25	DLG4	0.003779	LYN	-0.003200
26	C14orf1	0.003745	BTK	-0.003056
27	JAK1	0.003667	JAK3	-0.003018
28	PIAS1	0.003654	YAP1	-0.002875
29	FOS	0.003410	GSK3B	-0.002822
30	PLCG1	0.003333	ATM	-0.002806
31	CHD3	0.003285	YWHAQ	-0.002480
32	EWSR1	0.003279	BCL2	-0.002338
33	PIK3R1	0.003136	WAS	-0.002229
34	NFKBIA	0.003024	PPP2R5A	-0.002188
35	PML	0.002953	MUC1	-0.002134
36	PRNP	0.002944	SUV39H1	-0.002114
37	RBPMS	0.002917	TGFBR2	-0.002039
38	CCND3	0.002851	ADAM15	-0.001992
39	RUNX2	0.002838	MAGEA11	-0.001903
40	MAP2K1	0.002709	POU2F1	-0.001759
41	PSEN1	0.002696	SYN1	-0.001755
42	BRCA1	0.002660	PRKCG	-0.001745

Continued on next page

Supplementary Table 1 – continued from previous page

Rank	Gene	$\Delta\kappa_{OS} > 0$	Gene	$\Delta\kappa_{OS} < 0$
43	XPO1	0.002648	RGS2	-0.001736
44	DVL2	0.002554	DNM2	-0.001634
45	CRK	0.002550	PAK1	-0.001629
46	TRAF6	0.002531	FEZ1	-0.001536
47	MCM7	0.002298	JAK2	-0.001488
48	NEDD4	0.002287	UPF1	-0.001487
49	RAD51	0.002286	MDM2	-0.001449

Top 50 genes ranked by positive ($\Delta\kappa_{OS} > 0$) and negative ($\Delta\kappa_{OS} < 0$) difference in average scalar curvature between alive ($n = 13$) and dead ($n = 32$) cohorts (at last follow-up).

Supplementary Table 2: Changes in average scalar curvature based on sample type (primary (P) vs metastasis (M))

Rank	Gene	$\Delta\kappa_{PM} > 0$	Gene	$\Delta\kappa_{PM} < 0$
0	TP53	0.077029	ESR1	-0.036440
1	GRB2	0.052007	SMAD3	-0.021618
2	ATXN1	0.047675	CDKN1A	-0.020347
3	PRKCA	0.042002	JUN	-0.015349
4	CREBBP	0.026753	SRC	-0.013494
5	SMAD2	0.019927	LYN	-0.011455
6	AKT1	0.017599	MDFI	-0.010594
7	TGFBR1	0.012515	EGFR	-0.008569
8	SMAD4	0.011964	MAPK14	-0.008072
9	CTNNB1	0.010307	CCNE1	-0.007612
10	EWSR1	0.010187	GSK3B	-0.006226
11	VIM	0.009960	RB1	-0.005719
12	EP300	0.009048	RUNX2	-0.005594
13	APP	0.007988	CCND3	-0.005412
14	HSP90AA1	0.007883	MYOC	-0.005080
15	MYC	0.007121	JAK2	-0.004539
16	PTK2	0.005757	CDKN1B	-0.004367
17	HGS	0.005709	DAXX	-0.004313
18	YWHAE	0.005597	RBPMS	-0.004050
19	DLG4	0.005540	PIK3R2	-0.004026
20	SVIL	0.005213	MAP3K7	-0.003988
21	COIL	0.005048	PRKCD	-0.003983
22	PIK3R1	0.004457	MDM2	-0.003608
23	ITGB1	0.004411	NCOA2	-0.003404
24	STAT1	0.004319	RAF1	-0.003399
25	SLC9A3R1	0.004226	RAC1	-0.003303
26	MAPK1	0.004204	AKT2	-0.003245
27	ABL1	0.003941	PLCG1	-0.002819
28	CDK5	0.003806	HRAS	-0.002807
29	BRCA1	0.003210	IGF1R	-0.002795
30	ACTN1	0.003170	EZR	-0.002769
31	RANBP9	0.003063	MLLT4	-0.002732
32	PRKCG	0.002898	PRKCB	-0.002682
33	GFI1B	0.002779	POU2F1	-0.002679
34	TLE1	0.002757	FASLG	-0.002679
35	CHD3	0.002688	JAK1	-0.002676

Continued on next page

Supplementary Table 2 – continued from previous page

Rank	Gene	$\Delta\kappa_{PM} > 0$	Gene	$\Delta\kappa_{PM} < 0$
36	CCND1	0.002670	GNAI1	-0.002552
37	SYK	0.002652	PCNA	-0.002510
38	MAPK8	0.002550	PLG	-0.002244
39	DVL2	0.002504	FN1	-0.002154
40	YAP1	0.002367	XPO1	-0.002072
41	BCL2	0.002288	ACTB	-0.002056
42	HTT	0.002270	CDC42	-0.002046
43	POLR2A	0.002198	SHC1	-0.001900
44	TRIP13	0.002189	SUMO4	-0.001896
45	ATM	0.002159	JAK3	-0.001888
46	CRMP1	0.002127	COPS5	-0.001875
47	ACTG1	0.002125	CD247	-0.001809
48	C14orf1	0.002115	PIK3CA	-0.001768
49	SUMO2	0.001974	RXRG	-0.001731

Top 50 genes ranked by positive ($\Delta\kappa_{PM} > 0$) and negative ($\Delta\kappa_{PM} < 0$) difference in average scalar curvature between P ($n = 13$) and M ($n = 32$) groups.

Supplementary Table 3: **Changes in average scalar curvature with respect to the reference topology**

Rank	Gene	$\Delta\kappa_{ref} > 0$	Gene	$\Delta\kappa_{ref} < 0$
0	TP53	0.143999	SRC	-0.070044
1	EP300	0.089752	ATXN1	-0.041866
2	AR	0.055047	PTK2	-0.040546
3	TGFBR1	0.050407	MYC	-0.032654
4	MAPK1	0.041837	LYN	-0.026977
5	ESR1	0.025883	SHC1	-0.021819
6	PIK3R1	0.024060	GSK3B	-0.017265
7	SMAD3	0.023103	JUN	-0.015936
8	RB1	0.021967	PCNA	-0.015054
9	EWSR1	0.020344	PLCG1	-0.014421
10	SMAD4	0.019587	MAPK14	-0.014055
11	SMAD2	0.019196	CDKN1A	-0.011164
12	CREBBP	0.018241	YWHAQ	-0.008725
13	ABL1	0.016583	MDM2	-0.007339
14	CSNK2A2	0.012132	NCOA2	-0.007238
15	DLG4	0.011312	CCNE1	-0.006786
16	CTNNB1	0.010916	CDKN1B	-0.006716
17	BRCA1	0.010490	HCK	-0.006688
18	EGFR	0.009949	PAK1	-0.006402
19	YWHAE	0.009881	DAXX	-0.006289
20	GFI1B	0.009811	HSF1	-0.006140
21	ACTB	0.009696	BCL2L1	-0.005700
22	RAC1	0.009329	RBL1	-0.005566
23	MAGEA11	0.009010	MDFI	-0.005182
24	BTK	0.008727	STAT1	-0.004959
25	XRCC6	0.007943	ACVR1	-0.004607
26	UBB	0.007720	SUMO1	-0.004323
27	AKT1	0.007642	RANBP9	-0.004323
28	CHD3	0.007446	COPS5	-0.004273

Continued on next page

Supplementary Table 3 – continued from previous page

Rank	Gene	$\Delta\kappa_{ref} > 0$	Gene	$\Delta\kappa_{ref} < 0$
29	TLE1	0.007259	CDK5	-0.004024
30	SAT1	0.006644	MYOC	-0.003970
31	JAK2	0.006454	FN1	-0.003823
32	DVL2	0.006230	PARP1	-0.003753
33	SYK	0.006148	CDK4	-0.003741
34	NOTCH1	0.005997	CCND3	-0.003324
35	POLR2A	0.005812	COPS6	-0.003277
36	NCOR1	0.005568	SMURF1	-0.003160
37	HSP90AA1	0.005143	HIPK2	-0.003070
38	INSR	0.005123	POU2F1	-0.003066
39	CRK	0.004940	XPO1	-0.003044
40	PRKCB	0.004893	TGM2	-0.003020
41	BCAR1	0.004520	FGFR1	-0.002781
42	HTT	0.004499	PRNP	-0.002770
43	BCL2	0.004241	MUC1	-0.002713
44	SH3KBP1	0.003904	TRAF6	-0.002684
45	UTP14A	0.003815	YAP1	-0.002674
46	PRKCD	0.003753	MCM2	-0.002534
47	RASA1	0.003679	RUNX2	-0.002534
48	RARA	0.003616	PRSS23	-0.002533
49	FXR2	0.003610	NINL	-0.002514

Top 50 genes ranked by positive ($\Delta\kappa_{ref} > 0$) and negative ($\Delta\kappa_{ref} < 0$) difference between average HGSOC scalar curvature ($n = 45$) κ_{HGS} and the scalar curvature of the reference topology κ_{top}. $\Delta\kappa_{ref} = \kappa_{HGS} - \kappa_{top}$.

Supplementary Table 4: **Intersection of 171 top ranked candidate genes listed alphabetically**

ABL1	CTNNB1	MAP2K1	PRKCE	SYK
ACTB	DAXX	MAP3K7	PRKCG	SYN1
ACTG1	DLG4	MAPK1	PRNP	TGFBR1
ACTN1	DNM2	MAPK14	PRSS23	TGFBR2
ACVR1	DVL2	MAPK8	PSEN1	TGM2
ADAM15	EGFR	MCM2	PTK2	TLE1
AKT1	EIF2AK2	MCM7	RAC1	TP53
AKT2	EP300	MDFI	RAD51	TRAF6
APP	ESR1	MDM2	RAF1	TRIP13
AR	EWSR1	MLLT4	RANBP9	UBB
ARRB2	EZR	MUC1	RARA	UPF1
ATM	FASLG	MYC	RASA1	UTP14A
ATXN1	FEZ1	MYOC	RB1	VIM
AXIN1	FGFR1	NCOA2	RBL1	WAS
BCAR1	FN1	NCOR1	RBPMS	XPO1
BCL2	FOS	NEDD4	RGS2	XRCC6
BCL2L1	FXR2	NFKBIA	RHOA	YAP1
BRCA1	GFI1B	NINL	RPA1	YWHAE
BTK	GNAI1	NOTCH1	RUNX2	YWHAQ
C14orf1	GRB2	NR3C1	RXRG	
CASP8	GSK3B	NTRK1	SAT1	
CCND1	HCK	PAK1	SH3KBP1	
CCND3	HDAC3	PARP1	SHC1	
CCNE1	HGS	PCNA	SLC9A3R1	
CD247	HIPK2	PDPK1	SMAD2	
CDC42	HRAS	PIAS1	SMAD3	
CDK4	HSF1	PIK3CA	SMAD4	
CDK5	HSP90AA1	PIK3R1	SMAD7	
CDKN1A	HTT	PIK3R2	SMARCA4	
CDKN1B	IGF1R	PLCG1	SMURF1	
CHD3	INSR	PLG	SNAPIN	
COIL	ITGB1	PML	SRC	
COPS5	JAK1	POLR2A	STAT1	
COPS6	JAK2	POU2F1	SUMO1	
CREBBP	JAK3	PPP2R5A	SUMO2	
CRK	JUN	PRKCA	SUMO4	
CRMP1	LYN	PRKCB	SUV39H1	
CSNK2A2	MAGEA11	PRKCD	SVIL	

Supplementary Table 5: 100 identified candidate genes based on risk listed alphabetically

ACTB	ACVR1	ADAM15	AKT1	APP
AR	ARRB2	ATXN1	AXIN1	BCL2
BRCA1	BTK	CASP8	CD247	CDC42
CDK5	CDKN1A	CHD3	COIL	COPS6
CREBBP	CRK	CRMP1	CSNK2A2	CTNNB1
DLG4	DVL2	EGFR	EIF2AK2	EP300
ESR1	EWSR1	FASLG	FGFR1	FN1
FXR2	GNAI1	GRB2	GSK3B	HDAC3
HGS	HIPK2	HRAS	HSF1	HSP90AA1
HTT	JAK1	JUN	LYN	MAGEA11
MAPK1	MAPK14	MDM2	MUC1	MYC
MYOC	NCOR1	NR3C1	NTRK1	PAK1
PARP1	PCNA	PDPK1	PIK3R1	PIK3R2
PLCG1	POLR2A	POU2F1	PPP2R5A	PRKCA
PRKCD	PRKCE	PTK2	RAC1	RAF1
RANBP9	RASA1	RB1	RHOA	RPA1
SHC1	SMAD2	SMAD3	SMAD4	SMAD7
SMARCA4	SMURF1	SNAPIN	SRC	STAT1
SUMO1	SUMO4	TGFBR1	TP53	UBB
VIM	XPO1	XRCC6	YWHAE	YWHAQ

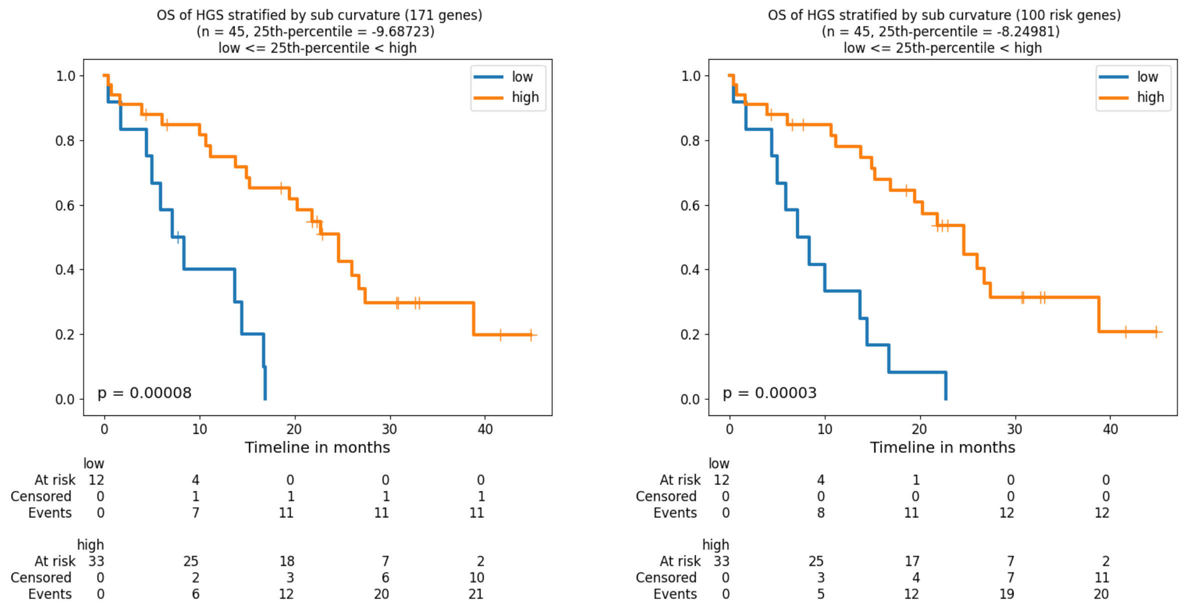

(a) (b)

Supplementary Figure 10: **Sub-curvature survival analysis.** Survival curves for HGSOC patients (n=45) based on the 25th percentile of sub-curvature over the following subsets of genes: (a) all 171 curvature identified genes (Supplementary Table 4) (b) 100 risk identified genes (Supplementary Table 5)

Sub-curvature survival analysis

The Kaplan-Meier analysis suggests that total curvature as a network measure of functional robustness is a more effective indicator of survival in HGSOC treated with ICIs than other genomic parameters. However, we expected that not all genes in the network were necessary to predict survival. We therefore defined the *sub-curvature* κ_g for any subset of genes g to be the sum of scalar (node) curvatures over all genes in the given subset:

$$\kappa_g = \sum j \in g\kappa_j. \tag{1}$$

We then repeated the survival analysis replacing total curvature with sub-curvature using the following two subsets of genes: (1) the *curvature identified genes* consisting of the 171 unique genes top-ranked by the various network curvature criteria (Supplementary Tables 1,2,3,1) listed alphabetically in Supplementary Table 4 and (2) the *risk identified genes* consisting of the top-ranked 100 genes by the curvature risk criterion (Table 1) listed alphabetically in Supplementary Table 5. Survival curves based on the 25th-percentile of sub-curvature values for each of these subsets are shown in Supplementary Figure 10. In both cases, the p-value (171 curvature identified genes: $p = 0.00008$; 100 risk identified genes: $p = 0.00003$) is about 1 order of magnitude smaller as compared to the total curvature, suggesting that the curvature methodology identifies key genes pertaining to survival.

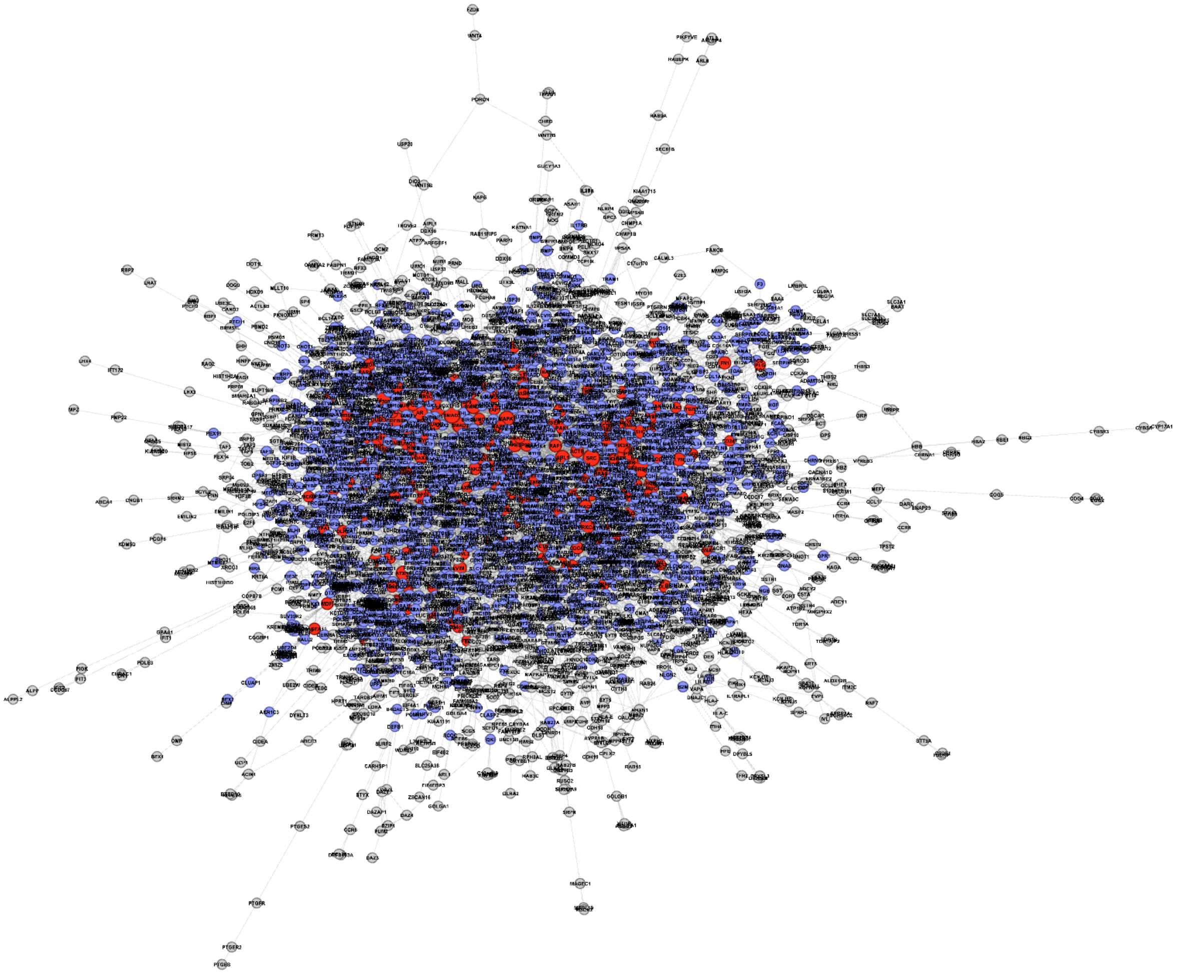

Supplementary Figure 11: **Network visualization.** Full resulting network taken as the largest connected component of the intersection between the HPRD and data (3,489 nodes, 9,710 edges, average degree = 5.57) is shown in ForceAtlas 2 layout using Gephi [46]. Top 171 identified genes are shown in red. Neighbors of the identified genes are shown in blue. Node size is scaled by degree.

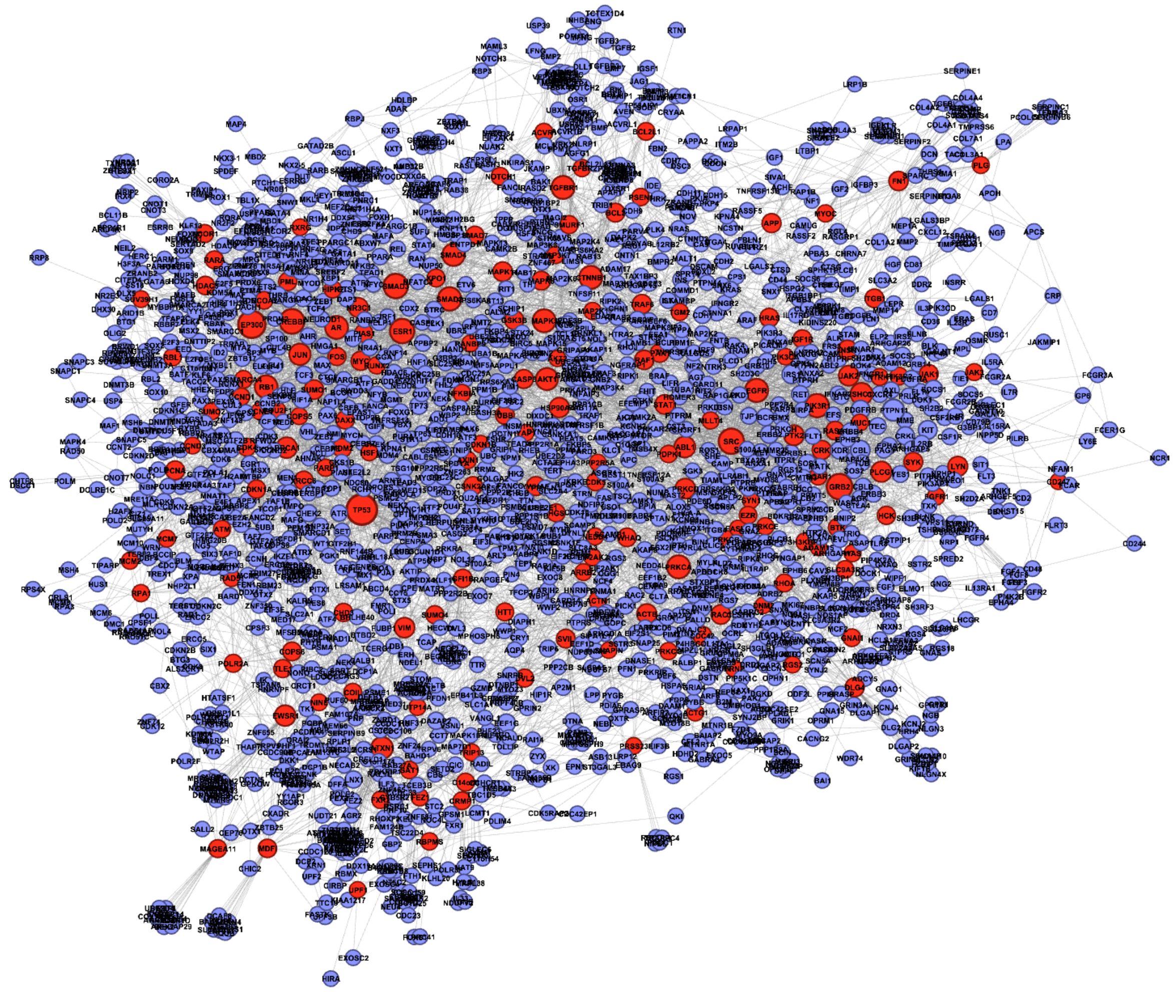

Supplementary Figure 12: **Subnetwork consisting of top 171 identified genes (red) and their neighbors (blue).** This subnetwork has 2,084 nodes in total, 7,359 edges and average degree = 7.06. Nodes are scaled by degree and the configuration was generated using Gephi's ForceAtlas 2 layout [46].

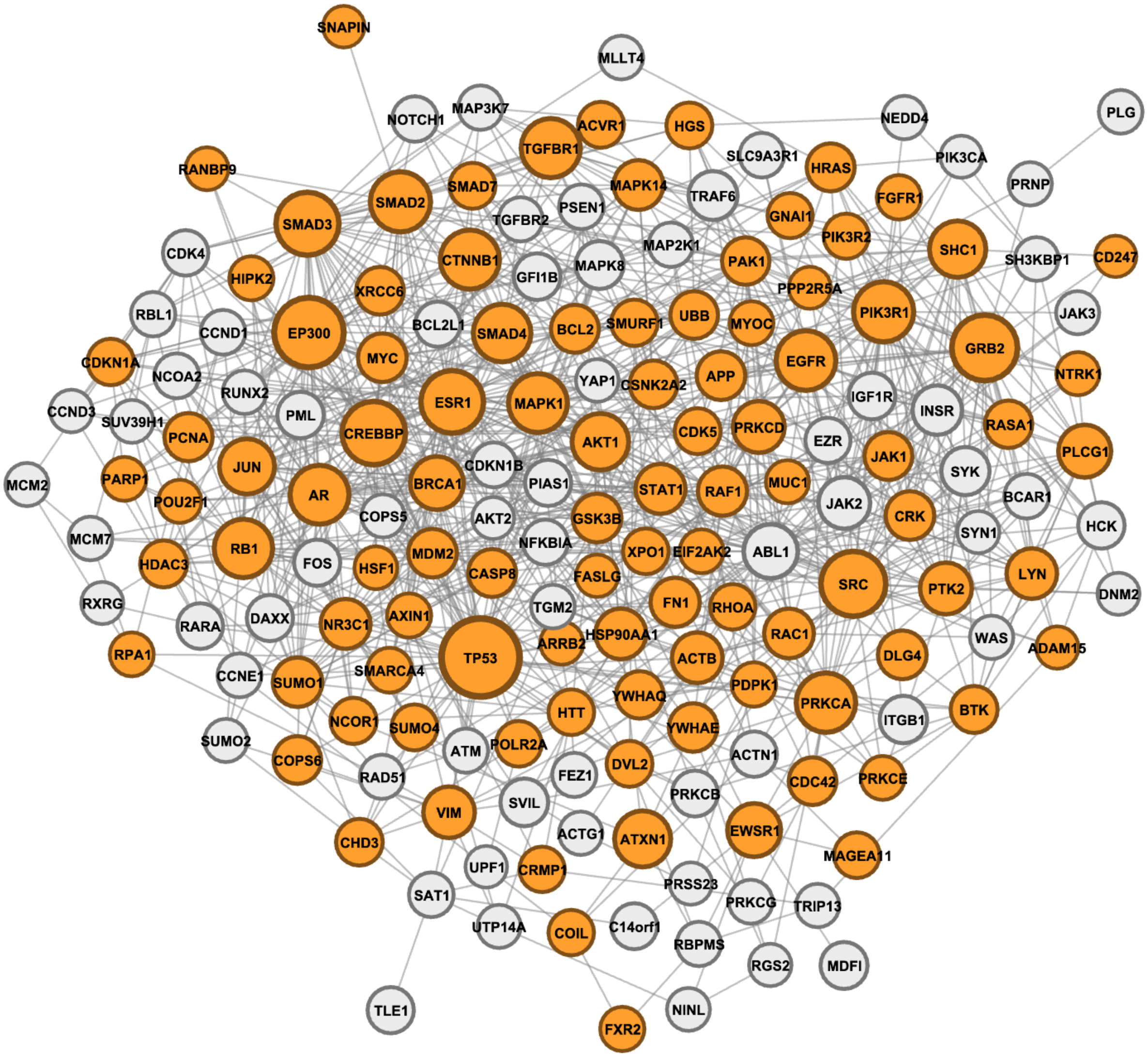

Supplementary Figure 13: **Subnetwork consisting of top 171 identified genes.** The 100 risk associated genes are shown in orange. Nodes are scaled by degree and the configuration was generated using Gephi's ForceAtlas 2 layout [46].

Method validation on Metabric data

For further validation, the methodology was applied to the Metabric dataset, a larger and publicly available data set with $n = 1903$ samples. The resulting Kaplan-Meier analysis and log-rank p-value are shown in Supplementary Figure 14.

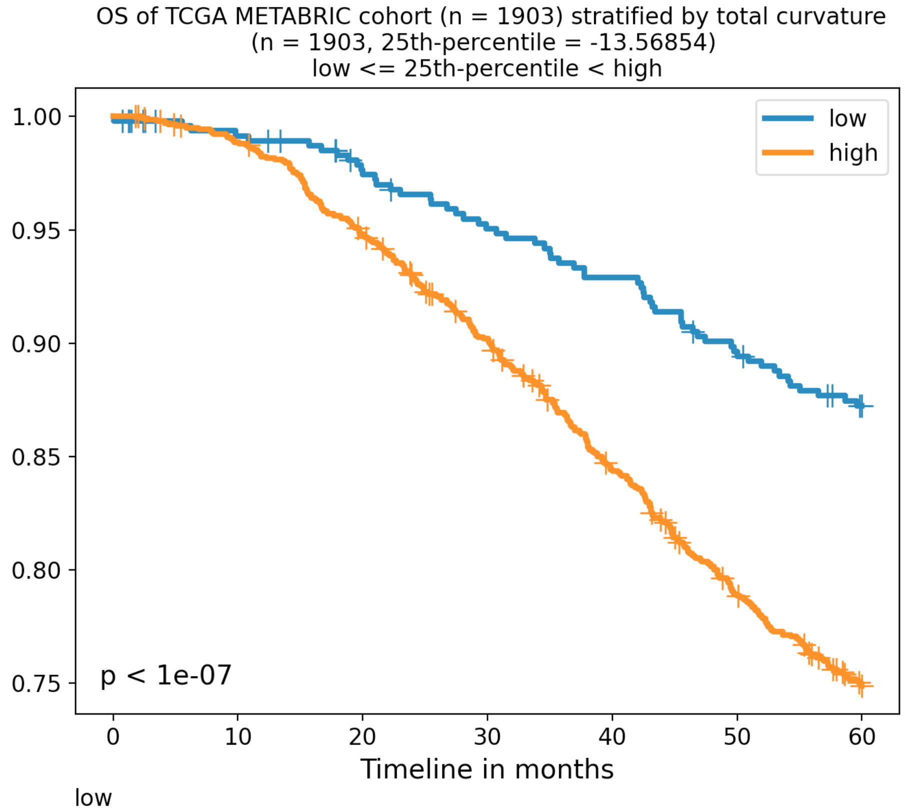

**low**							
At risk	476	465	451	439	429	412	0
Censored	0	7	13	14	14	15	417
Events	0	4	12	23	33	49	59
**high**							
At risk	1427	1405	1345	1271	1182	1100	0
Censored	0	6	8	17	24	30	1074
Events	0	16	74	139	221	297	353

Supplementary Figure 14: **Kaplan-Meier survival analysis of the Metabric dataset.** The cohort ($n = 1903$) was stratified into low and high groups by the 25th percentile of total curvature with the p-value derived from the log-rank test.
